# Antimicrobial photodynamic therapy against oral biofilm: influencing factors, mechanisms, and combined actions with other strategies

**DOI:** 10.3389/fmicb.2023.1192955

**Published:** 2023-06-09

**Authors:** Yijun Li, Guanwen Sun, Jingchan Xie, Suli Xiao, Chen Lin

**Affiliations:** ^1^Department of Endodontics, Stomatological Hospital of Xiamen Medical College, Xiamen, China; ^2^Department of Stomatology, Fujian Medical University Xiamen Humanity Hospital, Xiamen, China

**Keywords:** antimicrobial photodynamic therapy, reactive oxygen species, oral biofilms, mechanism, combination therapy

## Abstract

Oral biofilms are a prominent cause of a wide variety of oral infectious diseases which are still considered as growing public health problems worldwide. Oral biofilms harbor specific virulence factors that would aggravate the infectious process and present resistance to some traditional therapies. Antimicrobial photodynamic therapy (aPDT) has been proposed as a potential approach to eliminate oral biofilms via *in situ*-generated reactive oxygen species. Although numerous types of research have investigated the effectiveness of aPDT, few review articles have listed the antimicrobial mechanisms of aPDT on oral biofilms and new methods to improve the efficiency of aPDT. The review aims to summarize the virulence factors of oral biofilms, the progress of aPDT in various oral biofilm elimination, the mechanism mediated by aPDT, and combinatorial approaches of aPDT with other traditional agents.

## Introduction

Oral infectious disease, such as caries, endodontic, periodontitis, and peri-implantitis, impact an increasing population and pose a great threat to the healthcare and economy worldwide. As acknowledged, the origination of those diseases is influenced by an endogenous dysbiosis of the oral microbial communities but not by the introduction of exogenous microorganisms into the oral ecosystem (Lamont et al., 2018). The driven factors, including host diet, oral hygiene, and antibiotic consumption, could alter the microbial composition and the behavior of the microbial communities, then transform into a pathogenic state and lead to the initiation of infection (Li et al., 2022). The dental biofilm can be used to describe the collective activities and interactions of microbial communities that reside on various surfaces of hard tissue and soft tissue in the mouth, which is particularly participated in the pathogenesis of oral infectious diseases. Biofilm is termed as “the coaggregation of microbes that adhere to biotic and abiotic surfaces are embedded in self-produced exopolysaccharide matrix (EPS)” since 1985. In analogous to other biofilms in the host body, the notorious oral biofilms are not easily treatable through the existing antimicrobial strategies, since the refractory biofilm results from complex physical and biological properties with multiple cell signaling pathways, and also often involves multi-species interactions. Antimicrobial photodynamic therapy (aPDT) refers to the combined reaction of photosensitizer (PS) and appropriate wavelength of light source in the oxygen microenvironment. It is proposed that aPDT could be an efficient adjunctive approach in the disinfection of oral infectious diseases. This review aims to provide a concise overview of the existing literature on aPDT in oral biofilm, including the virulence factors of oral biofilm, the efficacy of aPDT on different oral biofilms, the involved mechanism of aPDT in biofilm inactivation, and the combined application of aPDT with other antimicrobials.

### The unique oral biofilm and structure influence antimicrobial efficacy

**Figure 1:**
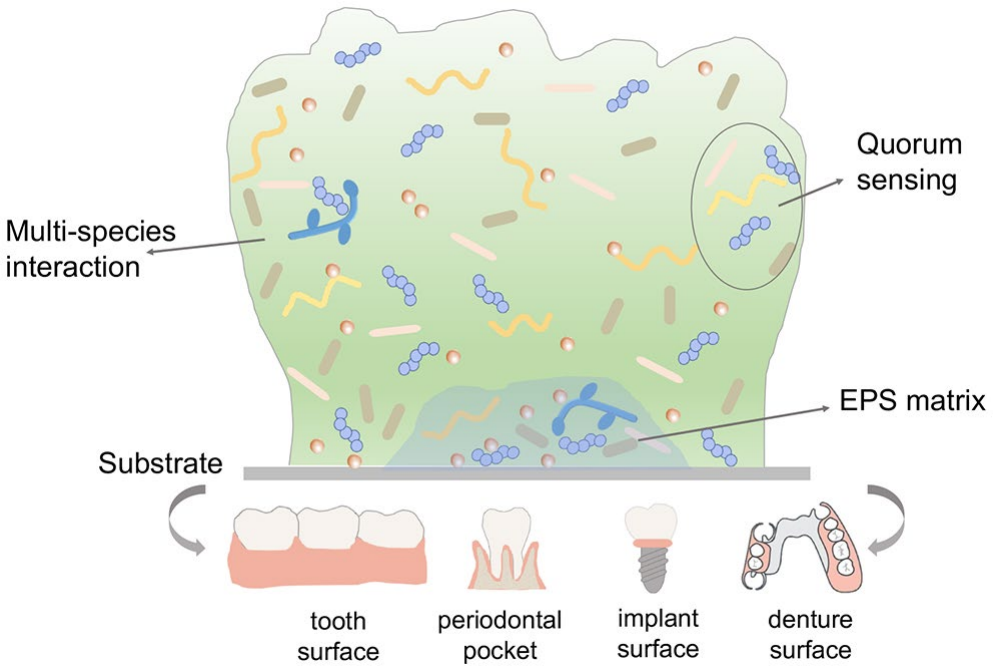
Schematic description of multiple virulence factors involved in oral biofilms.

The oral microorganisms adhere to the tooth surface or restorations successfully, start to proliferate and form micro-colony, and subsequently initiate biofilm formation. The altered virulence traits in biofilm compared to planktonic cultures protect microorganisms against antimicrobials. Figure 1 depicts the virulence factors of oral biofilm. Understanding how these virulence factors contribute to biofilm formation process and oral microbial dysbiosis is crucial to develop new treatment strategies for oral infectious diseases.

#### Extracellular polymeric substance

EPS was originally thought to be mainly extracellular polysaccharides but was redefined, as it became obvious that it also consists of proteins, nucleic acids, lipids and other biopolymers. It is well known that EPS accounts for 90% weight of biofilm, which serves as an adhesion site for microorganism aggregation, a nutrition source for microorganisms, a protective barrier to host defenses, and a reservoir of water against desiccation, those functions enable the biofilm to harbor a different lifestyle from planktonic counterparts (Flemming and Wingender, 2010; Cugini et al., 2019). Besides, the EPS matrix can retain virulent chemicals secreted by microbes and create pathogenic environments (such as acidic pH and hypoxia) that contribute to the progression of the disease. Many oral microorganisms are able to produce abundant EPS, among which *streptococcus mutans* (*S. mutans*) have been identified as the typical rich-produced EPS microorganism in the oral cavity. Its remarkable ability in producing matrix makes it to be a dominant cariogenic bacterial species. *Prevotella intermedia* (*P. intermedia*) is known to produce viscous exopolysaccharide that consists of mannose (Kwack et al., 2022). The presence of EPS in *P. intermedia* enables it to result in more severe periodontal abscesses in mice and resist the internalization of human polymorphonuclear leukocytes (Yamanaka et al., 2011). Thus, the strategies for the elimination of oral biofilm could target degrading EPS structure, inhibiting EPS synthesis, blocking the adhesion between EPS and the microbial surfaces, and regulating the EPS metabolism-related gene expression (Lin et al., 2021; Zhang et al., 2021).

#### Quorum sensing

Quorum sensing refers to a process that microbial cells orchestrate collective behaviors through sensing cell density and then altering gene expression. The microbes could communicate with each other by producing, detecting and responding to extracellular signaling molecules. There are some types of quorum sensing systems reported to date in oral microbes, including ComD/ComE two-component signal transduction system in *S. mutans*, the peptide-based Fsr quorum sensing systems in *Enterococcus faecalis* (*E. faecalis*), and farnesol in *Candida albicans* (*C. albicans*) (Senadheera and Cvitkovitch, 2008; Polke et al., 2018; Ali et al., 2022). The quorum sensing system plays a particularly essential role in bioluminescence, sporulation, specific gene expression, virulence factors secretion as well as biofilm organization. Apart from controlling microbial virulence, quorum sensing has been confirmed to be related to antimicrobial resistance (Wang Y. et al., 2019; Huang et al., 2020). Recently, the regulation effect of quorum sensing on efflux pump expression has been demonstrated (Subhadra et al., 2020). The efflux pumps are integral membrane proteins located in the cell membrane of bacteria, which could expel drugs out of membrane and thereby reduce the drug concentration that leads to antimicrobial resistance. Since quorum sensing is crucial for the various process of biofilm formation, the inhibition and interference of those communication processes have been proposed as important possibilities in the control of biofilm.

#### Multi-species interaction

The human oral cavity is an ecosystem of diverse organisms that successfully coexist and proliferate through continuous adaptation and coordinated responses to other co-colonizing organisms, diet changes, and other environmental stresses. These features of oral biofilm suggest that microbes may express complicated intraspecific and /or interspecific interaction mechanisms to fit the residing environmental fluctuations. The researchers have identified the microbial species and investigated the physiology of microbes from clinical samples via cultivation-dependent methods and high-throughput sequencing (Sedghi et al., 2021; Wade, 2021). The most common interactions in multi-species are synergistic, antagonistic, and competitive. Cooperation among biofilm communities could be intraspecific or interspecific, whose interaction often results in several beneficial phenotypes, including cross-feeding metabolic interaction, the stimulating effect on biofilm formation, and elevated resistance to antimicrobials or host immune defense system. It has been demonstrated that the cross-kingdom relationship between *S. mutans* and *C. albicans* promotes biofilm formation, elevates cariogenic potential and increases tolerance to antimicrobials (Sztajer et al., 2014; Kim et al., 2018). In another study, Xu et al. demonstrated that concomitant infection of *Streptococcus oralis* (*S. oralis*) and *C. albicans* increased the frequency and size of oral thrush lesions in a rat model (Xu et al., 2017). Other interactions of the multi-species biofilm in the oral ecosystem have been extensively revealed in previous studies, which is reported to be a pivotal role in increasing the disease risk and protective mechanisms against host immune response (Satala et al., 2021; Souza et al., 2022). The oral infection is a consequence of polymicrobial biofilm, which requires antimicrobials should be effective against various pathogens.

#### Biofilm resistance

In the past decade, government statements and scientific reports have revealed the seriousness of antimicrobial drug resistance and called for new movements in infection control concerning the alarming death rate due to antimicrobial resistance (Wall, 2019). That is, human has entered the post-antibiotic era where people would not survive unpredictable infections that could not be controlled by antibiotics. The underlying mechanisms of antimicrobial resistance are listed elsewhere including a reduction in membrane permeability, alteration of the target site, activation of efflux pumps, restriction of antibiotic access, and synthesis of enzymes for inactivation of antibiotics (Brauner et al., 2016). Though previous studies have rarely mentioned the emergence of resistant microorganisms after antimicrobial uses in the mouth, and those antiseptics are considered to be relatively safe, the accumulating evidence has shown some oral microorganisms present resistance towards chlorhexidine (Brookes et al., 2021; van de Lagemaat et al., 2022). Schwartz and co-workers have examined the phenotypic adaption of bacteria upon repeated exposure to oral antiseptics (Schwarz et al., 2021). Their data suggested that phenotypically adapted strains decrease the susceptibility toward benzalkonium chloride, chlorhexidine (CHX), or cetylpyridinium chloride after repeated sub-inhibitory exposure. Another randomized controlled clinical trial reported that local application of minocycline results in a transient selection of resistant bacterial species to minocycline in subgingival plaque samples and saliva (Teles et al., 2021). These results lead to concerns about the potential resistance development of oral microorganisms towards oral antiseptics.

Due to these hallmarks, the biofilm is difficult to amend, can reoccur after a period of clinical intervention, and become persistent. Though antibiotic is a frequent option for many infections, however, they could not be the preferred choice in the treatment of oral infectious disease due to the unique structure of the oral cavity and restorative materials, and low concentration of the drug after administration. Thence, the control and prevention of biofilm formation is still an arduous task with the few existing antimicrobial approaches available. Researchers have been trying to extend the arsenal of therapeutic options in oral biofilm-associated infection.

#### Antimicrobial photodynamic therapy (aPDT) area

**Figure 2:**
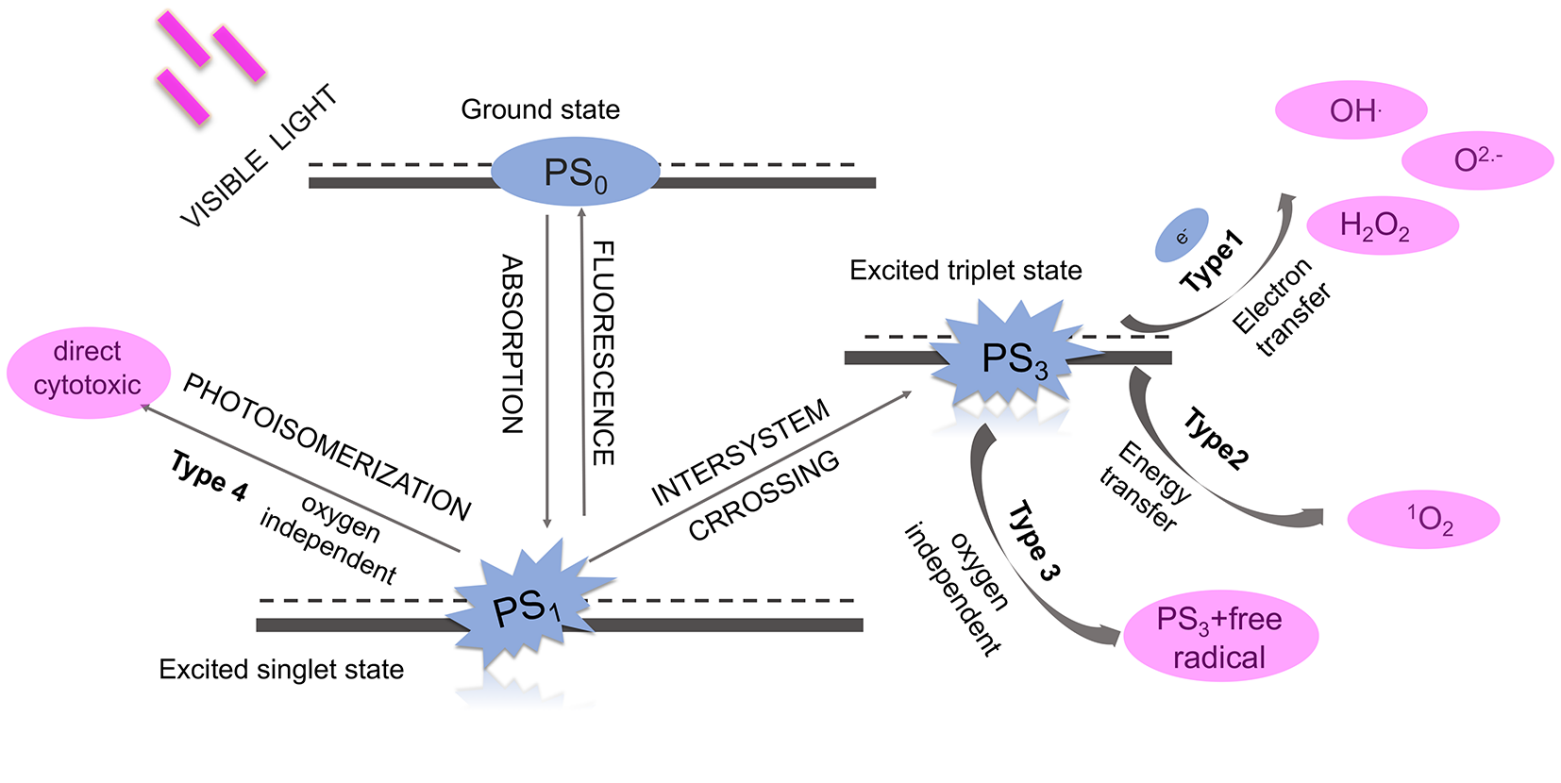
The photochemical mechanisms of photodynamic therapy illustrated in previous studies.

The idea of introducing aPDT into antimicrobial areas dates back to the 1970s when a scientist applied the combination of proflavine and light to treat experimental herpetic keratitis in rabbit (Moore et al., 1972). This approach has since attracted considerable attention from scientists, until the early 21st century, researchers defined photodynamic therapy targeting microorganisms as antimicrobial photodynamic therapy. aPDT uses a non-toxic substance called PS to produce reactive oxygen species (ROS) in the irradiation of light. It is accepted that, unlike antibiotics, aPDT could be an effective approach in the treatment of infectious diseases from multiple targets (Wainwright et al., 2017). Figure 2 shows the four mechanisms of photodynamic actions. The detailed mechanism of aPDT can be described as follows, the ground state PS (S_0_) would enter into a short-lived and unstable state (called the first excited singlet state, S_1_) after irradiating with a light source in the proper wavelength. To keep stable, the S_1_ PS could return to the ground state via emitting fluorescence or internal conversion with energy lost. Alternatively, it also could undergo intersystem crossing which involves changes in electron spin to convert into an excited triplet state (S_3_). The S_3_ PS would initiate the following two classical pathways, both of which are dependent on oxygen. The Type I mechanism involves electron-transfer reactions from triplet state PS to the biomolecules to form superoxide, hydroxyl radical (•OH), and hydrogen peroxide (H_2_O_2_). The Type II mechanism includes energy-transfer reactions from triplet state PS to the ground state molecular oxygen to generate singlet oxygen (^1^O_2_) (Huang et al., 2012b). In addition, two other oxygen-independent mechanisms have been proposed. Type III mechanism refers to a photoactivation process independent of oxygen and efficient for inactivating anaerobic microbes, leading to DNA damage in microbial cells (Warrier et al., 2021). While Type IV mechanism refers to a photoisomerization reaction that leads to the structural change from S_1_ state (Scherer et al., 2017). Type IV mechanism could directly activate the PS and induce cell death without any need for secondary oxygen-dependent reactions (Scherer et al., 2017).

#### Characteristics of ROS

Different types of ROS may correspond to different mechanisms of action. Superoxide is a negatively charged free radical that only oxidizes thiols, ascorbate, and catecholamines. As a primary radical, it can be reduced into secondary radical species like H_2_O_2_ and hydroxyl radicals (Van Acker and Coenye, 2017). However, superoxide could not penetrate the cell membrane so it only reacts where it is produced (Wang et al., 2018). H_2_O_2_ is a neutral molecule that could penetrate the cell membrane easily and participate in the Fenton reaction in the presence of ferrous iron. It is reported that H_2_O_2_ could attack microorganisms by inducing DNA damage without altering the integrity of the cell (De Furio et al., 2017). •OH is one of the deleterious ROS and can not be scavenged by enzymatic reactions. The formation of hydroxyl radical depends largely on the metabolism-related NADH depletion, the tricarboxylic acid (TCA) cycle, the electron transport chain, damage to the iron sulphur clusters in proteins, and stimulation of the Fenton reaction (Kohanski et al., 2007). In contrast to superoxide and H_2_O_2_, •OH could react readily with cellular components, including DNA, proteins, lipids and carbohydrates. ^1^O_2_ is a highly chemically reactive substance that could attack many essential cellular macromolecules, resulting in microbial death. Though highly reactive, it has a short lifespan due to its unstable electronic configuration, which hinders its continuous oxidation effect.

#### PS type and light source

The selection of an appropriate and effective PS is crucial to the antimicrobial outcomes of aPDT. So far, various natural and chemically synthesized photosensitizing agents have been applied in the PDT field, mainly highly conjugated unsaturated organic molecules. Generally, these PSs have higher absorption coefficients in the visible light region, preferably in the near-infrared region, to ensure the effective penetration of light into tissue. However, PSs used for anti-infection are a bit different from those for anti-tumor in terms of delivery rate and removal method. The ant-tumor PS was injected intravenously to reach the tumor area, which makes the uptake of PS slow. The ant-infection PS is delivered locally or by instillation, then diffusion into the matrix and bound with microbial cells by charge–charge interactions. As gram-negative and gram-positive bacteria have negatively charged moieties, cationic photosensitizers bind more easily to their cell envelope and further initiate the photoactivated reaction. The binding is essential for the inactivation of gram-negative bacteria whose outer membrane is too thick for PS molecules to penetrate. Cationic PS like phthalocyanine, methylene blue (MB), or toluidine blue O (TBO) shows excellent results in killing *Escherichia coli* (*E. coli*) and *Pseudomonas aeruginosa* (*P. aeruginosa*). Though the presence of cationic charge is one of the important factors in final antimicrobial effectiveness, the hydrophilic moieties should be considered in the synthesis of PS in order to prevent aggregation in aqueous media. Some literatures have encapsulated water-soluble PSs into nanostructured materials like chitosan, liposome or micelles to enhance water solubility, thus improving an excellent biocompatibility and antimicrobial activity. Due to the unique characteristics of oral biofilm and the different structures of oral microorganisms, the desirable PS in the treatment of oral infection should possess the following properties: no cytotoxicity to surrounding tissues, excellent stability after local delivery and not degraded by matrix, high affinity and selectivity to various microbes, high quantum yield of ROS.

There are three main light sources in the aPDT field, including laser, light-emitting diode (LED) and halogen lamps. Laser irradiation has some advantages over other light sources like monochromaticity and high intensity, enabling the shortening of irradiation time but still delivering a required dose. However, the monochromaticity and high-cost limit the usage of lasers in the clinic. In contrast to laser, LED have become popular for aPDT due to their low cost, ease of manipulation and access to tissue surfaces. Besides, LED has a broader range of wavelengths that could excite multiple PSs within its emission spectrum at a time. The advantage of the halogen lamp is that it can be spectrally filtered to match any photosensitizer. However, compared with laser or LED, it has poor directivity and monochromaticity, low intensity and insufficient energy. Halogen lamps cannot be effectively coupled to fiber bundles or liquid optical guides, so they will generate heat during irradiation.

The dosimetric parameters are key components that determine the efficacy of aPDT treatment during clinical practice. Accurate light dose measurement is extremely important during aPDT operation. There are four methods for PDT dosimetry, including implicit dosimetry, biophysical/biological tissue response monitoring, explicit dosimetry, and direct dosimetry (Kim and Darafsheh, 2020). The explicit measurement of light dosimetry is challenging since it depends on factors such as PS concentration, ground-state oxygen, and the delivered dose. It is proposed that the measurement of singlet oxygen can reflect the direct PDT dosimetry. The time-resolved measurement of ^1^O_2_ luminescence is the most commonly used method for determining ^1^O_2_ lifetimes and quantum yields (Lin et al., 2011). Other indirect measurement methods of ^1^O_2_ mainly utilize electron spin resonance (ESR) or optical probes to react with ^1^O_2_, and then detect ESR spectral signals, fluorescence signals, or chemiluminescence spectral signals, respectively.

#### aPDT efficiency on various oral biofilms

In addition to being effective in killing both oral gram-positive bacteria and gram-negative bacteria in suspension, aPDT is also demonstrated to be active against oral fungus. Owing to the different lifestyles between the planktonic state and biofilm state, this section focuses on the researches of aPDT efficiency on biofilms.

#### Carious biofilm

Table 1 presented the studies regarding the effects of aPDT on carious biofilm in recent five years. The existence of tooth decay is a direct consequence of mineral loss and the result of the combined activities of biofilms formed by acidic/aciduric microorganisms. *S. mutans* is closely linked to the occurrence of caries due to its several typical caries-related virulence traits, such as biofilm formation capacity, acid production, genetic competence advantage, and stress tolerance ability. Therefore, many studies have chosen *S. mutans* biofilm as a model to assess the effectiveness of new antimicrobials on carious-related biofilm. The phenphenothiazinium dyes including MB and TBO have been extensively studied in aPDT studies against *S. mutans* biofilm (de Sousa Farias et al., 2016; Mendez et al., 2018; Tokubo et al., 2018). These results reported that TBO or MB-mediated aPDT could yield significant elimination of *S. mutans* biofilm at different ages, resulting in bactericidal reduction ranging from 1 log_10_ to 5 log_10_. Apart from phenphenothiazinium dyes, curcumin, indocyanine green (ICG) and chlorin e6 (Ce6) have been used as PSs in several works (Cusicanqui Méndez et al., 2018; Kim et al., 2020). Nie et al. demonstrated that, with the same concentration of PS and light dose, Ce6-mediated aPDT elicits more significant bactericidal activity than MB-aPDT (with an excess of 3 log_10_ killing) (Nie et al., 2020). In addition to biofilm elimination ability, a previous study has demonstrated that aPDT could influence the virulence traits of *S. mutans* (Pourhajibagher et al., 2022).

**Table 1:** The list of the findings of the relevant studies regarding the effect of aPDT on carious biofilm.

Photosensitizer (concentration)	Light source	Microorganism	Biofilm growth/substrate	Viability loss	Reference
Fotoenticine (200 μg/mL)	660 nm, LED 30 J/cm^2^	dentin caries sample of adults	120 h/Bovine Tooth	2.1–2.8 log_10_ for streptococci, 1.8–3.2 log_10_ for lactobacilli, 1.7–3.2 log_10_ for yeasts	Garcia et al. (2021)
ICG (2 mg/mL)	808 nm, diode laser 300 mW, 18 J	*S. mutans*	24 h/HA	2.25 log_10_	Kim et al. (2020)
MB, Ce6 (25, 50, 100, 200 μM)	660 nm, LED 15 J/cm^2^	*S. mutans*	48 h/Glass coverslips	MB ≈ 2 log_10_ (25, 50, 100 μM) Ce6 ≈ 5 log_10_ (200 μM)	Nie et al. (2020)
HpIX HpIXDC	420–480 nm, blue LED, 75 J/cm^2^	*S. mutans*	48 h/Metallic or ceramic brackets	Metallic HpIX = 0, HpIXDC = 2.43 log_10_ Ceramic HpIX =0, HpIXDC =3.6 log_10_	Lacerda Rangel Esper et al. (2019)
TBO (50 μM)	670 nm, diode laser, 65w	*S. mutans* *L. casei* *A. viscosus* *S. salivarius*	24 h/Tooth slice	*S. mutans =* 81% *L. casei =* 86.71% *A. viscosus =* 48%, *S. salivarius =* 14%	Darmani et al. (2018)
MB (100 μM)	660 nm, LED 10, 40, 60, 65, 75, 80 J/cm^2^	*S. mutans*	72 h/96-well plate	MB ≈ 2 log_10_ (10 J/cm^2^) MB ≈ 5 log_10_ (40 J/cm^2^) MB ≈ 11 log_10_ (60, 65, 75, 80 J/cm^2^)	Leal et al. (2017)
MB (100 μg/mL)	630 nm LumaCare™ 56.6, 149, 317.05, 475.58 J/cm^2^	*S. mutans*	120 h/ HA	75 J/cm^2^ 1.58 log_10_ for streptococci, 1.36 log_10_ for *S. mutans*, 1.97 log_10_ for *lactobacilli* 37.5 J/cm^2^ 0.66 log_10_ for streptococci, 0.72 log_10_ for *S. mutans*, 0.77 log_10_ for *lactobacilli*	Mendez et al. (2018)
Cur (600 μM)	450 nm, blue LED 0, 37.5, 75 J/cm^2^	Dentin caries sample of children	120 h/ Bovine dentin slice	75 J/cm^2^ 1.52 log_10_ for streptococci, 1.70 log_10_ for *S. mutans*, 2.33 log_10_ for *lactobacilli* 37.5 J/cm^2^ 1.71 log_10_ for streptococci, 1.68 log_10_ for *S. mutans*, 1.79 log_10_ for *lactobacilli*	Cusicanqui Méndez et al. (2018)
TBO (100 μg/mL)	638.8 nm, red laser, 65w	*S. mutans*	120 h/HA	56.6 J/cm^2^ ≈ 1.5 log_10_ 56.6 J/cm^2^ ≈ 2 log_10_ 317.05 J/cm^2^ ≈ 2.38 log_10_ 475.58 J/cm^2^ ≈ 2.27 log_10_	Aragao et al. (2019)
Cur (10,50,100 μg/mL) DAC (10,50,100 μg/mL)	440–480 nm, LED 1.2 J/cm^2^	*S. mutans*	24 h/96-well plate	100 μg/mL CUR: 8.9 log_10_ 100 μg/mL DAC: 8.7 log_10_	Sanches et al. (2019)
TBO (50,100, 150 μg/mL)	667 nm, LED 36, 108, 180 J/cm^2^	*S. mutans*	48 h/96-well plate	36 J/cm^2^ ≈ 1 log_10_ 108 J/cm^2^ ≈ 1.5–2.5 log_10_ 180 J/cm^2^ ≈ 3.5 log_10_ (100,150 μg/mL)	Balhaddad et al. (2020)
TBO (100 μg/mL)	630 nm, LED 31.5 J/cm^2^	*S. mutans* *L. acidophilus*	48 h/ Bovine dentin slices	*S. mutans*≈1.5 log_10_ *L. acidophilus* ≈3.5 log_10_	Wu et al. (2019)

ICG, indocyanine green; MB, methylene blue; HpIX, hematoporphyrin IX; HpIXDC, hematoporphyrin IX dihydrochloride; Cur, curcumin; TBO, toluidine blue O; DAC, Diacetylcurcumin; LED, light emitting diode; HA, hydroxyapatite.

It is acknowledged that testing new antimicrobial agents or protocols should be conducted on biofilm models pertinent to the clinical situation as possible. Using *S. mutans* as a single inoculum to develop biofilms may not fully represent the carious biofilm in the oral cavity, since the interaction between microorganisms also plays an essential part role in caries development. Quishida et al. established biofilm of *C. albicans*, *Candida glabrata (C. glabrata)*, and *S. mutans, and used* photodithazine and LED to evaluate the efficacy of aPDT on multi-species biofilm (Quishida et al., 2015). The results revealed that 0.2 mg/mL of photodithazine in association with 37.5 J/cm^2^ led to a decrease of 1.21 log_10_, 1.19 log_10_, and 2.39 log_10_ in the amounts of *C. albicans*, *C. glabrata*, and *S. mutans*, respectively. Karygianni et al. recruited six healthy volunteers to wear upper jaw acrylic devices with bovine enamel slabs for 2 h and 3 days to form early and mature biofilm *in situ* (Karygianni et al., 2014). Then *in situ* biofilms were subjected to 100 μg/mL TBO or Ce6 in combination with visible light plus water-filtered infrared-A. They showed that TBO or Ce6-mediated aPDT induce a microorganism reduction of more than 99.9% against initial oral biofilm, and Ce6-mediated aPDT presented higher effectiveness in eradicating 89.62% of 3-day biofilm bacteria compared to TBO--mediated aPDT (82.25%). These results helped us understand that aPDT is sufficient to disinfect monospecies biofilms and multi-species biofilm.

#### Endodontic biofilm

The complete elimination of biofilms and their byproducts from the root canal system is essential to successful endodontic treatment. Previous studies verified root canal systems, especially in some anatomical variations, remain untouched after conventional mechanical debridement (Wu et al., 2003; Del Carpio-Perochena et al., 2011). Sodium hypochlorite (NaClO), the most effective irrigation solution, can penetrate the dentinal tubules by 100 μm to exert a bactericidal effect (Kuga et al., 2011). However, pathogenic microorganisms’ invasion depth reaches up to 1,000 μm. In an attempt to further promote the cleaning of the root canal, calcium hydroxide and other drugs are used for the intra-canal medication (Shuping et al., 2000). While studies have demonstrated the existence of microorganisms after using calcium hydroxide as an intra-canal medication drug for more than 1 week (Sathorn et al., 2007). Furthermore, long-term application of calcium hydroxide dressing leads to a decrease in microtensile fracture strength of endodontically treated teeth, causing a risk of root fracture (Batur et al., 2013). Based on the dilemma of root canal disinfection, many technologies such as aPDT have been introduced to make up for deficiencies of current antimicrobial strategies during root canal treatment to facilitate complete disinfection. Table 2 presented the studies regarding the effects of aPDT on endodontic biofilm in recent 5 years.

**Table 2:** The list of the findings of the relevant studies regarding the effect of aPDT on endodontic biofilm.

Photosensitizer	Light source	Microorganism	Biofilm growth/substrate	Reduction in microbial load	Reference
TBO, MB (0.1 mg/mL) Cur (1 mg /mL) ICG (5 mg/mL)	MB-aPDT: 660 nm, diode laser, 117.18 J/cm^2^ TBO-aPDT: 635 nm, diode laser, 171.87 J/cm^2^ ICG-aPDT: 810 nm, diode laser, 171.87 J/cm^2^ Cur-aPDT: 450 nm, LED, 300–420 J/cm^2^	*E. faecalis*	24 h/12-well plate	Cur-aPDT: 99.6% ICG-aPDT: 98.2% TBO-aPDT: 85.1% MB-aPDT: 65.0%	Pourhajibagher et al. (2018c)
Cur (40 mM) ICG (1 mg/mL)	ICG-aPDT: 810 nm，diode laser, 250 mW Cur-aPDT: 450 nm, LED, 1000–1,400 mW/cm^2^	*E. faecalis*	24 h/Flat-bottomed microplates	Cur-aPDT: 83.6% ICG-aPDT: 75.2%	Pourhajibagher et al. (2018b)
ICG (1 mg/mL)	ICG-aPDT: 810 nm，diode laser, 31.2 J/cm^2^	*E. faecalis*	14 d/Human single-rooted teeth	ICG-aPDT: 81.6%	Bolhari et al. (2018)
MB (100 μg/mL)	660 nm, diode laser, 150 mW, 6 J	*E. faecalis*	21 d/Single-rooted teeth	98.8%	Asnaashari et al. (2019)
MB (100 μg/mL)	660 nm, diode laser, 150 mW, 6 J	*E. faecalis*	21 d/Human single-rooted teeth	Apical: 90.7% Coronal: 56%	Armand et al. (2019)
TB (0.1 mg/mL)	810 nm, diode laser, 200 mW	*E. faecalis*	7 d/Human single-rooted teeth	2.615 log_10_	Armand et al. (2019)
Riboflavin (0.1 mg/mL)	810 nm, diode laser, 100 mW, 100 HZ	*E. faecalis, S. aureus and C. albicans mixed biofilm*	15 d/Human single-rooted teeth	99.9999977%	Katalinic et al. (2019)

*E. faecalis* is frequently isolated from persistent root canal infections since it could penetrate deeper dentinal tubules, survive in harsh conditions, and form resistant biofilms (Stuart et al., 2006; Zhang et al., 2015). Numerous investigations have reported the effectiveness of aPDT on *E. faecalis* biofilm *in vitro*, among them, the most studied PSs are MB, TBO, ICG, and curcumin (Cur) (Lopez-Jimenez et al., 2015; Pourhajibagher et al., 2018c; Moradi et al., 2021). These reports have demonstrated the efficacy of aPDT in significant bacteria elimination and biofilm disruption. However, *E. faecalis* is not always presented in clinical failure cases and is also not a prevalent species in the root canal system. The over-attention paid to *E. faecalis* biofilm elimination is likely to have misled the evaluation of the true effectiveness of aPDT on root canal biofilm. To reproduce the clinical scenario of root canal treatment, Hoedke et al. investigated the effectiveness of adjunctive aPDT treatment following irrigations against a 5-day multi-species biofilm on root canals. The multi-species biofilm consists of *E. faecalis*, *S. oralis*, and *P. intermedia*, which were isolated from one infected root canal (Hoedke et al., 2017). The immediate reduction of adjunctive use of aPDT on bacteria counts in the root canal was not different from that of the control group (without treatment). But the bacterial load was reduced with adjunctive aPDT after 5 days of further incubation when compared to the control group. Another study recruited two volunteers to wear the Hawleys orthodontic device with dentin blocks for 72 h to form an oral biofilm (Rosa et al., 2017). Then the specimens were treated with MB-mediated aPDT or in combination with NaClO or CHX and live/dead assay was used to analyze the microbial viability. Although aPDT resulted in a microbial reduction, the residual counts of microorganisms were still high and the effect was unsatisfactory. But when in combination with common irrigations, aPDT could highly aid NaClO and CHX in microorganism eradication. These results were in line with prior studies, suggesting that aPDT might act synergistically with irrigations in root canal disinfection.

The application of aPDT in root canal disinfection is different from the way for cavity disinfection. Without underlying dentin, PS could directly contact the periodontal ligament cells of the root apex through the apical foramen during dark incubation. Upon illumination, the generated ROS may damage the cells residing in root apex. Several reports have investigated the potential cytotoxicity of aPDT and found that the cytotoxicity is minor and acceptable (Gomes-Filho et al., 2016; Strazzi-Sahyon et al., 2022). However, the PS types and cell lines involved in these researches were too insufficient to assess the true cytotoxicity. Therefore, the evaluations of aPDT cytotoxicity should be conducted from *in vitro* and *in vivo* in order to establish a safe and effective protocol that can be reproduced in the clinical situation.

In summary, the biofilm models used in these studies vary in terms of substrate, biofilm growth condition, and biofilm age. So far, there is no standard method of biofilm model construction for the assessment of antimicrobial agents or techniques in root canal disinfection. Additionally, the sampling, the experimental groups, and the methods of evaluating antimicrobial efficiency are quite different. Thus, it seems unreasonable to draw the conclusion of the superiority in performance of aPDT over other techniques from pairwise comparisons of the above studies. However, a large portion of researchers supports the supplementary role of aPDT in root canal disinfection. The recognitions of bactericidal activity and minimal invasive property of aPDT allow aPDT accessible in one single visit treatment and some failed endodontic cases.

#### Periodontitis and peri-impalntitis biofilm

Periodontitis is an inflammatory process occurring around the tooth with the gradual bleeding of the gingival and progressive destruction of supporting bone. The supragingival and subgingival biofilm adhering to the tooth is the initiating factor of periodontitis. Similar to periodontitis, peri-implantitis refers to pathological inflammatory reactions in implant-surrounding soft and hard tissues due to microbial composition dysbiosis, which could lead to bone resorption and decreased osseointegration (Mombelli et al., 2012). Thus, adequate microbial biofilm elimination from the tooth surface or implant surface is an essential step in the treatment of periodontitis and peri-implantitis before any indicated regenerative surgical procedures. Mechanical scaling, systemic antibiotic therapy and local antiseptic application are the main methods in the non-surgical treatments of periodontitis and peri-implantitis, however, these approaches alone are limited in surface debridement and complete elimination of pathogenic microbes (Renvert et al., 2008; Figuero et al., 2014). Recently, aPDT has been suggested as an adjunct to non-surgical mechanical debridement for the treatment of periodontitis and peri-implant disease in several systemic reviews (Sculean et al., 2021). Table 3 presented the studies with regard to the effects of aPDT on endodontic biofilm in recent 5 years.

**Table 3:** The list of the findings of the relevant studies regarding the effect of aPDT on periodontal biofilm.

Photosensitizer	Light source	Microorganism	Biofilm growth/substrate	Reduction in microbial load	Reference
TBO (0.4 mg/mL)	630 ± 5 nm, LED, 40–60 J/cm^2^	*P. gingivalis*	24 h/24-well plate	*P. gingivalis*:1.03 log_10_ CFU	Ding et al. (2021)
Ce-6 (50 μM)	450 /660 nm, LED 15 J/cm^2^	Saliva-derived biofilm	48 h/glass coverslips	No change	Nie et al. (2021)
MB (10 mg/mL) FMN (0.18 mg/mL)	MB-aPDT: 670 nm, red diode laser, 75 mW/cm^2^ FMN-aPDT: 450–470 nm, LED, 3700-4,000 mW/cm^2^	*S. aureus*	48 h/SLA titanium discs	MB-aPDT: 1.228 log_10_ CFU FMN-aPDT: 1.234 log_10_ CFU	Leelanarathiwat et al. (2020)
ICG (50, 150, 300, 500 μg/mL)	570–1,400 nm, vis+wIRA, 60 J/cm^2^	plaque samples in RTF	NA	50 μg/mL = 1 log_10_ CFU 150, 500 μg/mL < 1 log_10_ CFU 300 μg/mL no effect	Solarte et al. (2022)
ICG (250 μg/mL)	810 nm, diode laser,100 J/cm^2^	*S. mutans*	1 d, 4 d /96-well plate	1d biofilm = 4.19 log_10_ CFU 4 d biofilm = 4.14 log_10_ CFU	Nikinmaa et al. (2020)
Ce6 (100, 200 mM)	450 nm/600 nm, LED 30 J/cm^2^	*S. oralis* *F. nucleatum* *P. gingivalis* *A. actinomycetemcomitans*	48 h/96-well plate	*S. oralis* 100 mM Ce-6 (450 nm) = 4.51 log_10_ 200 mM Ce-6 (450 nm) = 8.46 log_10_ 100 mM Ce-6 (660 nm) = 3.78 log_10_ 200 mM Ce-6 (660 nm) = 4.03 log_10_ *F. nucleatum* 100 mM Ce-6 (450 nm) = 0.67 log_10_ 200 mM Ce-6 (450 nm) = 0.96 log_10_ 100 mM Ce-6(660 nm) = 3.44 log_10_ 200 mM Ce-6 (660 nm) = 3.46 log_10_ *A. actinomycetemcomitans* 100 mM Ce-6 (450 nm) = 3.89 log_10_ 200 mM Ce-6 (450 nm) = 4.01 log_10_ 100 mM Ce-6 (660 nm) = 0.99 log_10_ 200 mM Ce-6 (660 nm) = 1.03 log_10_ *P. gingivalis* 100 mM Ce-6 (450 nm) = 8.24 log_10_ 200 mM Ce-6 (450 nm) = 8.24 log_10_ 100 mM Ce-6(660 nm) = 8.24 log_10_ 200 mM Ce-6 (660 nm) = 8.24log_10_	Garcia de Carvalho et al. (2020)
ICG (1 mg/mL)	810 nm, diode laser, 31.2 J/cm^2^	*P. gingivalis* *A. actinomycetemcomitans* and *P. intermedia* *mix-species biofilm*	72 h/ Implants	IGC-aPDT≈ 4 log_10_	Pourhajibagher et al. (2020b)
SAPYR (100 μM) SAGUA (100 μM)	380–660 nm, gas-discharge lam, 30 J/cm^2^	*A. naeslundii*, *F. nucleatum* and *P. gingivalis* mix-species biofilm	72 h/ 96-well palte	SAPYR-aPDT: *A. naeslundii =* 6 log_10_ *F. nucleatum =* 6.1 log_10_ *P. gingivalis =* 4.4 log_10_ SAGUA-aPDT *A. naeslundii =* 2.8 log_10_ *F. nucleatum =* 2.4 log_10_ *P. gingivalis =* 2 log_10_	Cieplik et al. (2018)
TBO (100 μg/mL)	635 nm, LED, 90 J/cm^2^ 635 nm, diode laser, 13.2 J/cm^2^	*A. actinomycetemcomitans*	48 h/SLA titanium disk	TBO-aPDT (LED) ≈ 2log_10_ TBO-aPDT (LASER) ≈ 2log_10_	Ghasemi et al. (2019)
TBO (100 μg/mL)	660 nm, red laser, 50 J/cm^2^	Subgingival plaque	15 d/ Implants	0.49 log_10_	Batalha et al. (2021)
Phycocyanin (125, 250, 500 μg/mL)	635 nm, diode laser, 220 mW	*A. actinomycetemcomitans*	48 h/SLA titanium disk	125 μg/mL:26.66% 250 μg/mL: 30.66% 500 μg/mL: 43.11%	Etemadi et al. (2020)
MB (100 μg/mL)	660 nm, diode laser, 100 mW	*S. aureus* *E. coli* *P. aeruginosa*	48 h/ Titanium disk	*S. aureus*<1 log_10_ *E. coli* ≈1.5 log_10_ *P. aeruginosa*<1 log_10_	Camacho-Alonso et al. (2022)
TBO (100 μg/mL)	635 nm, LED, 31.5 J/cm^2^	*S. aureus*	48 h/ Polished, SLA titanium disk	Polished ≈1 log_10_ SLA ≈ 1.5–2 log_10_	Cai et al. (2019a)
TBO (100 μg/mL)	635 nm, LED, 31.5 J/cm^2^	*P. gingivalis*	72 h/ Polished, SLA titanium disk	Polished≈1 log_10_ SLA ≈ 1 log_10_	Cai et al. (2019b)

FMN, flavin mononucleotide; SAPYR, 1-((1-Oxo-1H-phenalen-2-yl) methyl)-pyridinium chloride; SAGUA, 1-((1-Oxo-1H-phenalen-2-yl)methyl)-1-methyl-guanidinium chloride.

Due to similar features between periodontitis and peri-implantitis, many scholars support the concept that these two entities harbor identical microbial communities (Apatzidou et al., 2017). Prior studies have used probes targeting periodontal microbes specifically to identify most microbes within the peri-implant crevice. Therefore, *Porphyromonas gingivalis (P. gingivalis), Aggregatibacter actinomycetemcomitans* (*A. actinomycetemcomitans*), and *Tannerella forsythi* (*T. forsythi*) are commonly used oral microbiota for the studies of the antimicrobial strategy on periodontitis or peri-implant diseases (Persson and Renvert, 2014; Tzach-Nahman et al., 2017; Al-Ahmad et al., 2018). They can exist in the oral saliva and later transform into opportunistic pathogens to initiate the inflammatory response. Though the oral taxa that colonize around the implant site are more likely to originate from dental biofilm, some distinct differences were also found between periodontitis and peri-implantitis in microbiome composition (Kotsakis and Olmedo, 2021; Barbagallo et al., 2022). The difference may be due to the vast accumulation of metal ions, which exert an effect on microbiome profiles in the peri-implant sulcus (Safioti et al., 2017). Some gram-positive bacteria like *Staphylococcus aureus* (*S. aureus*) *and Staphylococcus epidermidis* (*S. epidermidis*), frequently identified in peri-implant sites, have a great affinity to the implant surface and produce β-Lactamase to inactive the antibiotics (Salvi et al., 2008; Burgers et al., 2019). *S. aureus* biofilm has become the most common *in vitro* model for assessing the effect of anti-infective therapy in peri-implantitis.

Earlier in the 1990s, two scholars found low-level helium–neon laser irradiation with dyes was effective in killing *P. gingivalis*, *Fusobacterium nucleatum* (*F. nucleatum*), and *A. actinomycetemcomitans*, emphasizing the potential role of aPDT in bacteria eradication (Dobson and Wilson, 1992). In contrast to other oral bacteria, there is one group of bacteria in periodontal pathogenic bacteria called black-pigmented bacteria, whose endogenous porphyrins could act as PS. Cieplik et al. treated *A. actinomycetemcomitans* suspension with 150 J/cm^2^ blue light without any dyes and observed a ≥ 5 log10 reduction in CFU amounts (Cieplik et al., 2014). In other studies, *P. gingivalis* and *F. nucleatum* were susceptible to blue light with a spectrum of 400–520 nm (Feuerstein et al., 2004; Yoshida et al., 2017). In addition to being effective against planktonic cultures, numerous *in vitro* studies have revealed that, for biofilms of these species, the combination of PS and light activation could lead to a reduction in viable counts, destruction in biofilm architecture, and disassembly of the biofilm matrix. The antimicrobial effectiveness of aPDT on these biofilms depends on PS type, light energy density, and biofilm maturity. The treatment of periodontitis is not only to remove biofilm and calculus via scaling but also to create a microenvironment conducive to periodontal tissue regeneration. Recent reports have explored the role of aPDT in the inflammatory cytokines in the periodontitis microenvironment. A recent investigation pointed out that some PSs could interact with LPS, modify the structure of LPS, and alter the biological effects in cells, which may provide a different insight into the treatments not only in dentistry but also in general health (Couto et al., 2021). The result was in line with the findings of Matsushima et al. (2021) who observed that aPDT treatment could lead to LPS decrease, organic resolution, organic debris removal, and root surface cleaning. Another study isolated inflamed human gingival fibroblasts (HGF) and treated them with MB plus light irradiation. The data showed that MB-mediated aPDT increases the expression of wounding healing genes including interleukin 6 (IL-6), type I collagen (COL1), fibronectin (FN), and basic fibroblast growth factor (bFGF) (Yang et al., 2020). The biological behaviors induced by aPDT are beneficial to HGF growth and conducive to wound healing in periodontitis.

Similar to periodontitis, the treatment of peri-implantitis is based on removing the biofilm debris from the implant surface in order to create a smooth and biocompatible surface for re-attachment. Previous studies have indicated that the appropriate combination of PS with a light source can result in a reduction in both counts and viability of both aerobic and anaerobic biofilms about peri-implantitis (Rismanchian et al., 2017; Azizi et al., 2018). The contemporary implants are designed with threads and nanostructured grooves to promote cellular osseointegration. Studies have used polished and SLA titanium disks or other titanium surfaces to establish biofilms and assessed the efficacy of aPDT on surface decontamination. Therefore, the studies have evaluated the effectiveness of new antimicrobials on biofilms established on different titanium surfaces. Cai et al. have investigated the efficacy of TBO-mediated aPDT on *P. gingivalis* and *S. aureus* biofilms on polished and SLA titanium disks and compared the effects of CHX on microbial reduction (Cai et al., 2019a,b). The authors showed that aPDT has a comparable effect as CHX in disinfecting biofilms, which was independent of the titanium surface. Considering that surface alteration has been observed in the implant surface following laser treatment, the study also observed the structure of polished and SLA titanium disk after aPDT treatment (TBO + 31.5 J/cm^2^ LED) and reported that aPDT does not affect titanium disk morphology. Apart from monospecies biofilm, blue light irradiation has been demonstrated to reduce bacteria counts and biofilm thickness in multi-species biofilm, especially for *P. gingivalis* and *T. forsythia* (Shany-Kdoshim et al., 2019). Meanwhile, aPDT showed potential in promoting gingival epithelial cells, gingival fibroblasts, and osteoblast-like cells attachment to dentin and titanium surfaces (Eick et al., 2017).

Despite the positive outcomes of aPDT efficacy in biofilm disinfection, the results of clinical trials are not as satisfying as *in vitro* data. There are several challenges during the use of aPDT in the periodontal pocket or peri-implant pocket disinfection. Firstly, gingival bleeding and inflammation will influence the efficiency of aPDT because proteins in serum or exudate can hamper photosensitizers uptake of tissue and gingival cervical fluid can dilute photosensitizers below the effective concentration. Secondly, the stratified keratinized gingival epithelium acts as a barrier that reduces the penetration depth of light illumination and utilization efficiency of aPDT. Thirdly, a periodontal microenvironment is associated with low oxygen tension due to inflammation progression. As aPDT is an oxygen-consumption process, the hypoxic periodontal pocket may limit the efficacy of aPDT. Therefore, it would be a great deal of work to be undertaken for the purpose of establishing the correct parameter for clinical practice and improving the aPDT effectiveness.

### Clinical trials of aPDT in oral infectious diseases

#### Caries

With respect to the clinical application, aPDT has dual utility for caries treatment. Table 4 listed some clinical trials of aPDT in the treatment of caries in recent five years. For those individuals who are prone to caries, aPDT could reduce microbial colonization and remove biofilm on the enamel surface, therefore controlling the incidence of tooth caries (Gomes-Filho et al., 2016; de Melo Soares et al., 2019). For instance, Melo et al. observed the reduction of cariogenic microbial load after the application of an aPDT procedure (Melo et al., 2015). Likewise, Clara et al. reported a dramatic decline in plaque samples after exposure to MB solution and irradiation with laser. These results suggested that aPDT could be an effective way to reduce carious pathogens both in saliva and plaque. Another alternative application of aPDT in caries is cavity disinfection during deep caries treatment. Following the notion of minimally invasive techniques, dentists employ selective removal of dental caries in order to avoid pulp exposure during cavity excavation, leaving the inner dentin layer untreated. It is reported that the hidden microorganisms in the inner can threaten the pulp viability and the success rate of caries filling. aPDT could be a promising method to reduce the microbial load in the inner layer after caries evacuation (Araujo et al., 2014). A clinical study has analyzed the use of aPDT as an adjunct for the partial removal of carious tissue (PRCT) of deciduous carious tissue (Bargrizan et al., 2019). The infected dentin was removed by drill and the remaining dentin was treated with aPDT. The clinical finding revealed that aPDT results in a significant reduction of *S. mutans* in salivary samples. However, there have been few clinical trials in relation to the application of aPDT in the caries field to date, which require more *in vivo* evidence to ensure clinical relevance.

**Table 4:** The list of the findings of the clinical studies regarding the effect of aPDT on carious lesions.

Type of disease	Study design	Groups	PS type and concentration	Light devices and dose	Follow-up period	Conclusion	Reference
Deep caries	RCT	Group 1: Caries removal with a low-speed drill Group 2: Partial removal of carious tissue + Papacarie ™ Group 3: Partial removal of carious tissue+ Papacarie ™ + *Bixa Orellana* extract Group 4: Partial removal of carious tissue + Papacarie ™ + *Bixa Orellana* extract+aPDT	NM	440-480 nm light-emitting diode, NM	Immediately 1 week, 1, 3, 6, and 12 month	Procedures using aPDT have been developed for the treatment of dental caries, but there are few controlled clinical trials in the literature confirming its efficacy.	Martins et al. (2023)
White spot lesions	RCT	Group 1: light-emitting diode Group 2: application of nanomicelle curcumin Group 3: aPDT Group 4: Fluoride anti-caries mouthwash	nanomicelle curcumin, 80 mg of 10 nm curcumin 10–15 min after breakfast for four months	450 ± 10 nm light-emitting diode, 200 mW	1, 2, 3, and 4 month	The antimicrobial and anti-virulence activities of Cur-aPDT against *S. mutans* were higher than the other treatment groups.	Hosseinpour-Nader et al. (2022)
Dental plaque *in vivo*	RCT	Control 1: no intervention Control 2: Erythrosine, no light Test 1: aPDT (continuous light) Test 2: aPDT (pulsed)	erythrosine-B, 220 μM	500–550 nm Tungsten filament lamp, 22.7 mW/cm^2^	2 week	Improving the clinical usefulness of PDT by reducing its overall treatment time seems to be promising and effective in killing *in vivo*-formed dental plaque biofilms.	Alsaif et al. (2021)
Dental caries lesion	RCT	Group 1: caries removal with a low-speed drill Group 2: application of aPDT with PapacarieMBlue.	PapacarieMBlue (addition of MB), NM	660 nm diode laser, 100 mW	3 month 6 month 12 month	Adding methylene blue dye to the formula of PapacarieMBlue might potentiate the antimicrobial action of aPDT and work more effectively on the infected dentin combined with a conservative, minimally invasive treatment.	Costa-Santos et al. (2019)
Severe early childhood caries	RCT	Group 1: Toluidine blue O Group 2: Laser Group 3: Toluidine blue O + Laser Group 4: no intervention	Toluidine blue O, 0.1 mg/mL	633 nm diode laser, 20 mW	1 day 3 day 1 week 2 week	aPDT modality may be used to decrease the colony count of salivary mutans streptococci in children with SECC.	Bargrizan et al. (2019)

RCT, randomized controlled trial; NM, not mentioned.

#### Endodontic infection

As acknowledged, mechanical instrumentation alone could not yield an adequate cleaning of the complex root canal system. In order to eliminate bacteria biofilm from the root canal system, many adjunctive approaches have been proposed to assist in the debridement of the root canal. aPDT has been indicated as a supplementary approach during root canal disinfection due to its extraordinary properties in inactivating various endodontic pathogens. Table 5 listed some clinical trials of aPDT in the treatment of endodontic infection in recent 5 years.

**Table 5:** The list of the findings of the clinical studies regarding the effect of aPDT on endodontic infection.

Type of disease	Study design	Groups	PS type and concentration	Light devices and dose	Follow-up period	Conclusion	Reference
chronic periapical periodontitis	RCT	Control: conventional endodontic treatment Test1:conventional endodontic treatment+ diode laser Test 2:conventional endodontic treatment+ aPDT	phenothiazinium chloride, 10 mg /mL	660 nm diode laser, 100 mW	Immediate	The results indicated that both aPDT and diode laser could be performed as adjuvants to standard endodontic treatment of the young permanent teeth with chronic periapical periodontitis.	Dragana et al. (2023)
Endodontic infection	RCT	Control: chemo-mechanical preparation Test: chemo-mechanical preparation+aPDT	methylene blue, 0.005%	Red laser, 30 mW	Immediate	Photodynamic therapy as an adjunct to CMP proved to be effective in improving root canal disinfection and reducing the LPS and LTA levels in teeth with primary endodontic infection.	Alves-Silva et al. (2023)
irreversible pulpitis or pulp necrosis	RCT	Control: mechanical–chemical preparation Test: mechanical–chemical preparation +aPDT	methylene blue, 0.005%	660 nm laser, 100 mW	1 month 3 month	Conventional treatment combined with antimicrobial PDT with parameters used in this study proved effective but presented equal efficacious capability to conventional endodontic treatment alone.	Okamoto et al. (2020)
Pulp Necrosis	RCT	Control: mechanical–chemical preparation Test: mechanical–chemical preparation +aPDT	methylene blue, 0.005%	660 nm laser, 9 J	2 month	aPDT did not promote better results in endodontic treatment, being similar to conventional treatment.	Moreira et al. (2021)
necrotic pulps and asymptomatic apical peri- odontitis	RCT	Control: root canal treatment Test: root canal treatment +aPDT+LLLT	methylene blue, 0.01%	660 ± 10 nm laser, 100 mW	7 day 14 day 30 day	Photobiomodulation had no significant effect on post-operative pain, tenderness, oedema and the use of analgesics after root canal treatment with foraminal enlargement, in single-rooted teeth treated in a single visit.	Guimaraes et al. (2021)

RCT, randomized controlled trial; CMP, chemo-mechanical preparation; LPS, lipopolysaccharide; LTA, lipoteichoic acid; LLLT, low-level laser therapy.

Garcez et al. verified that aPDT added to endodontic treatment considerably reduces bacterial load according to CFU counting assay (Garcez et al., 2015). Some studies reported the significant disinfecting effect of aPDT on infected root canals in single-visit endodontic treatment. For example, Rabello et al. demonstrated that the supplemental PDT was effective in reducing bacterial load in the one-visit, which could reduce the possibility of bacterial contamination during multiple visits (Rabello et al., 2017). In addition to enhancing bacteria reduction, aPDT has been evaluated for its efficacy on microflora resistant to previous antibiotic therapy. In a clinical trial by Garcez et al., they showed that conventional endodontic treatment leads to a significant decrease in amounts of microbial species, whereas the supplementary aPDT result in the elimination of drug-resistant species (Garcez et al., 2010). These results suggest that aPDT could be a promising approach adjunct to root canal disinfection especially for the teeth undergoing one-session endodontic treatment or retreatment.

aPDT also provides benefits in the endodontic treatment of primary teeth. For young patients who are poorly tolerant, the treatment of primary endodontic infection is quite complex. In addition, the presence of accessory foramens and ectopic root resorption also contributes to the complicacy of primary teeth treatment. It is proposed that it is necessary to use some other techniques and alternatives for microbial reduction in deciduous teeth (Pinheiro et al., 2009). A number of clinical trials have reported consistent results that aPDT favors a more reduction in microorganisms after following conventional chemomechanical instrumentation (Pinheiro et al., 2009; Okamoto et al., 2020). Another study has investigated whether the photobiomodulation effect of aPDT irradiation would relieve posto-perative pain after endodontic treatment (Guimaraes et al., 2021). Nevertheless, photobiomodulation with aPDT had no significant effect on post-operative pain.

#### Periodontitis

There are a number of published studies that investigate the adjunctive use of aPDT in the treatment of oral infectious diseases, especially periodontitis and peri-implantitis. Table 6 listed some clinical trials of aPDT in the treatment of periodontitis in recent 5 years. An ideal goal of periodontitis treatment would be the elimination of microbial biofilms and the regeneration of surrounding bone. However, basic mechanical approaches result in insufficient elimination of microbes, especially in deep periodontal pockets or furcation defects. Several adjunctive therapies that include local antibiotic delivery, antiseptics, and aPDT have been proposed to overcome the limitations. A previous study has assessed the effectiveness of aPDT monotherapy and scaling root planning (SRP) alone in improving clinical parameters of periodontitis (de Oliveira et al., 2007). The result all revealed that aPDT monotherapy is not as effective in the improvement of clinical parameters as SRP. Based on this finding, accumulating studies have examined the adjunctive role of aPDT. A meta-analysis of four RCTs indicated that adjunctive aPDT use shows a favorable effect compared to SRP alone in terms of clinical parameters including periodontal pocket and clinical attachment level (Xue and Zhao, 2017). The comparison between PDT and local antibiotic delivery has also been evaluated in the treatment of chronic periodontitis and aggressive periodontitis. Despite the encouraging results mentioned before, several clinical studies showed the adjunctive use of antibiotic use is more effective in periodontal pocket (PD) reduction, MMP-8 level decrease, and a low number of deep pockets than aPDT (Arweiler et al., 2014; Theodoro et al., 2017). However, the comparison of local antibiotic delivery or aPDT use is still datable since superior results of aPDT were also reported. Additionally, some studies have also validated the adjunctive role of aPDT in assisting SRP in aggressive periodontitis patients with higher levels of inflammation and low susceptibility to conventional periodontal treatments. For instance, a previous study has examined the efficacy of ICG-aPDT in the treatment of stage III grade C periodontitis in type-2 diabetes mellitus. Their follow-up results suggested a significant reduction in bacteria numbers and improvement in clinical parameters in the ICG-aPDT group compared to those without aPDT treatment.

**Table 6:** The list of the findings of the clinical studies regarding the effect of aPDT on periodontitis.

Type of disease	Study design	Groups	PS type and concentration	Light devices and dose	Follow-up period	Conclusion	Reference
Periodontitis	RCT	Control: FUMD Test: FUMD+ aPDT	ICG, 1 mg/mL	660 nm diode laser, 300 mW	Baseline 3 month 6 month	The combination of repeated ICG-aPDT and FMUD provided no benefits except for selective clinical and microbiological improvements compared to FMUD alone.	Annunziata et al. (2023)
Periodontitis	RCT	Control: root planning +scaling Test: root planing +scaling+aPDT	methylene blue, 100 μM	660 nm Laser, 100 mW	Baseline 3 month 6 month	The effect of methylene blue in surfactant did not cause enough phototoxic effects that could promote reduction of periodontal pocket depth.	Kassa et al. (2023)
Periodontitis	RCT	Control: scaling and root planing Test: scaling and root planing +aPDT	ICG, NM	909 nm diode laser, 300 mW	Baseline 3 month 6 month	Both treatments resulted in significant clinical periodontal improvements, but with no significant differences between groups except from inflammation parameters. aPDT using ICG resulted in significant reductions in BOP and PISA index, as well as in *P. gingivalis* and *A. actinomycetemcomitans* levels.	Costa et al. (2023)
Periodontitis	RCT	Control: scaling and root planning +aPDT Test: scaling and root planning + Er,Cr:YSGG laser	methylene blue, 10 mg/mL	660 nm InGaAs diode laser, 70 mW	Baseline 2 week 3 month	Adjunctive use of Er,Cr:YSGG laser with SRP showed better clinical outcomes than a-PDT with SRP.	Soundarajan and Rajasekar (2022)
Periodontitis	RCT	Control: aPDT Test: open flap debridement	methylene blue, 10 mg/mL	660 nm diode laser, 129 J/cm^2^，60s	Baseline 3 month 6 month	Open flap debridement was superior in reducing PPD in deep pockets compared to the aPDT. However, OFD resulted in greater GR. Both treatments lowered *P. gingivalis* levels but only Open flap debridement reduced levels of *A. actinomycemtemcomitans.*	Andere et al. (2022)
Periodontitis	RCT	Control: root surface debridement (RSD) Test: aPDT+RSD	ICG, 0.5 mg/mL	810 nm diode laser, 200 mW	Baseline 3 month 6 month	ICG-aPDT significantly improved clinical and antimicrobial parameters in well-controlled and poorly-controlled type-2 diabetes mellitus having stage III and grade C periodontitis. Glycemic status did not have negative impact in the reduction of periodontal parameters in either types of type-2 diabetes mellitus.	Al-Momani (2021)
Periodontitis	RCT	Control: SRP Test: aPDT+SRP	ICG, 0.1 mg/mL	808 nm diode laser, 200 mW	Baseline 1 month 3 month	A significant reduction in periodontal clinical parameters and microbial burden was seen in the aPDT group.	AlSarhan et al. (2021)
Periodontitis	RCT	Control: SRP+ Salvadora persica gel Test: SRP + aPDT	ICG, NM	810 nm diode laser, 100 mW	Baseline 3 month 6 month	Both the treatment modalities PDT and Salvadora persica gel helped in reducing periodontal inflammation. PDT reported significant gain in clinical attachment level, whereas the Salvadora persica gel significantly reduced the bleeding levels.	Niazi et al. (2020)
Periodontitis	RCT	Control: SRP Test: SRP + aPDT	Phenothiazine chloride, 10 mg/mL	660 nm diode laser, 70 mW	Baseline 2 week 1 month 3 month	No significant difference was seen in terms of clinical parameters between study groups.	de Melo Soares et al. (2019)
Periodontitis	RCT	Control: SRP Test: SRP + aPDT	Toluidine blue O, 0.1 mg/mL	635 nm diode laser, 200 mW	Baseline 3 month 6 month	The aPDT group, substantially reduced the inflammation, BOP, and compared to the control microorganisms burden of group.	Grzech-Lesniak et al. (2019)

RCT, randomized controlled trial; ICG, indocyanine green; FMUD, full-mouth ultrasonic subgingival debridement; SRP, subgingival root planing; RSD, root surface debridement; NM, not mentioned; BOP, bleeding on probing.

#### Peri-implantitis

Table 7 listed some clinical trials of aPDT in the treatment of peri-implantitis in recent 5 years. Peri-implantitis can be divided into peri-implant mucositis and peri-implantitis according to the severity of peri-implant tissue inflammation. Peri-implant mucositis refers to an inflammation of the mucous membrane surrounding the implant, while peri-implantitis is an inflammatory process affecting soft tissue and supporting bone. The consensus report of the sixth European Workshop on Periodontology stated that mechanical approach alone without other interventions is not effective for the treatment of peri-implantitis.

**Table 7:** The list of the findings of the clinical studies regarding the effect of aPDT on peri-implantitis.

Type of disease	Study design	Groups	PS type and concentration	Light devices and dose	Follow-up period	Conclusion	Reference
Peri-implantitis	RCT	Control: peri‐implant debridement Test: peri‐implant debridement+ aPDT	Chloroaluminum phthalocyanine, 0.005%	640 nm diode laser, 150 mW,	Baseline 3 month 6 month	Chloroaluminum phthalocyanine-mediated PDT proved effective in improving peri‑implant clinical outcomes and reducing cytokine levels in smoking patients with chronic hyperglycemia	Elsadek et al. (2023)
Peri-implantitis	RCT	Control: MD Test: MD + aPDT	ICG, NM	805 nm laser, 500 mW	Baseline 2 week 3 month	The application of PDT using 805‐nm laser and ICG as an adjunct therapy to MD did not provide any additional improvements in the clinical or biologic parameters of peri‐implant mucosal inflammation	Pourabbas et al. (2023)
Peri-implantitis	RCT	Control: MD Test: MD + aPDT	Chloroaluminum phthalocyanine	660 nm Laser, 100 mW	Baseline 3 month 6 month	Chloroaluminum phthalocyanine-assisted PDT helped to improve the clinical and cytokine levels after non-surgical peri‑implant mechanical debridement in treating peri‑implantitis patients in smokers.	Ahmed et al. (2023)
Peri-implantitis	RCT	Control: MD Test 1: MD+ aPDT (1 session) Test 2: MD + aPDT (2 sessions) Test 3: MD + aPDT (3 sessions)	Methylene blue, 0.005%	660 nm diode laser, 180 mW	Baseline 9 month	The use of aPDT as an adjunct to MD reduces the severity of peri‑implant mucositis but does not contribute towards bone regeneration in peri‑implant osseous defects.	Strazzi-Sahyon et al. (2022)
Peri-implant mucositis	RCT	Control: MD Test: MD + aPDT	Methylene blue, 0.005%	660 nm diode laser, 150 mW	Baseline 3 month	In the short term, a single session of aPDT as an adjunct to MD is effective in reducing peri-implant soft tissue inflammation and oral yeasts colonization in patients with peri-implant mucositis.	Shetty et al. (2022)
Peri-implantitis	RCT	Control: MD Test: MD + aPDT	ICG, 1 mg/mL	810 nm diode laser, 200 mW	Baseline 3 month 6 month	Multiple application of indocyanine-green mediated photodynamic therapy resulted in improved clinical and microbial parameters among type 2 DM subjects in the treatment of peri-implantitis.	Labban et al. (2021)
Peri-implantitis with abscess.	RCT	Control: MD + aPDT Test: MD+ amoxicillin and metronidazole	Methylene blue, NM	670 nm diode laser, NM	Baseline 6 month 12 month	PDT was equally effective in reducing severe peri-implant symptoms compared to antimicrobial therapy as an adjunct to mechanical debridement	Almohareb et al. (2020)
Peri-implantitis	RCT	Control: MD Test: MD + aPDT	methylene blue, 0.5 mg/mL	670 nm diode laser, 150 mW	Baseline 3 month 6 month	Adjunctive PDT helped in reducing the clinical peri-implant inflammation. However, no significant change was observed for biological bone biomarkers among tobacco smokers.	Al Deeb et al. (2020)
Peri-implant inflammation	RCT	Control: MD Test 1: MD + aPDT Test 2: MD+ antibiotics	Phenothiazine chloride, NM	660 nm diode laser, 100 mW	Baseline 6 weeks 12 weeks	This short term clinical study suggests that aPDT as an adjunct to MD is as efficacious as adjunctive antibiotic therapy. However, additional benefits in the reduction of bleeding scores were observed for aPDT in peri-implant inflammation among cigarette smokers.	Deeb et al. (2020)
Peri-implantitis	RCT	Control: subgingival sand-blasting Test: subgingival sand-blasting + aPDT	Toluidine blue, 10 mg/mL	635 nm light-emitting diode, 750 mW	Baseline 1 month 3 month 6 month	aPDT combined with mechanical debridement significantly improves clinical parameters in participants with peri-implantitis. Importantly, PDT achieved a better CAL than mechanical debridement and cleaning.	Wang H. et al. (2019)

RCT, randomized controlled trial; MD, mechanical scaling; ICG, indocyanine green; NM, not mentioned; CAL, clinical attachment loss.

Four RCTs have evaluated the adjunctive application of aPDT in the non-surgical treatment of peri-implant mucositis or peri-implantitis (Bassetti et al., 2014; Javed et al., 2017; Ahmed et al., 2020, 2023). All studies demonstrated adjunctive use of aPDT presents significant improvements for PD, bleeding on probing (BOP), and clinical attachment loss (CAL). With respect to bacteria count measurement, two studies have found that aPDT application, when compared to mechanical debridement, induced more reductions in the proportions of peri-implant pathogens (Labban et al., 2021; Shetty et al., 2022). These peri-implant pathogens include *P. gingivalis*, *Treponema denticola*, *P. aeruginosa*, and *S. aureus*. When comparing the effectiveness of aPDT versus local antibiotic delivery in four RCTs, all reports revealed no significance between these two therapies in improving clinical parameters. Apart from clinical, microbiological, and host-derived parameters, a few studies have investigated the effect of aPDT application on biological bone marker levels related to bone regeneration (ALHarthi et al., 2022; Strazzi-Sahyon et al., 2022). The finding appeared not to be consistent with some *in vitro* studies that have indicated the stimulating effect of aPDT on bone regeneration of mesenchymal stem cells.

## Mechanisms involved in aPDT procedure

**Figure 3:**
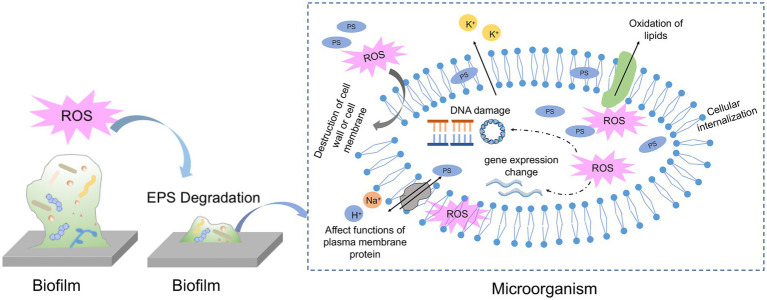
Schematic of proposed mechanisms of antimicrobial photodynamic therapy against biofilm and microbial cells.

So far, there is no consensus about the concrete binding location of PS to microbial cells or biofilms and the actual localization of ROS attack. For the assessment of the potential of aPDT, it is essential to find the impaired targets due to ROS generation in aPDT procedure. The specific ROS attack sites are influenced by multiple factors, such as PS characteristics, incubation time, and microbial species. Figure 3 has listed the proposed antimicrobial mechanism of aPDT procedure in biofilm and microbial killing.

### Degradation of EPS

As a natural physical barrier of biofilm, EPS comprises around 90% of the total biofilm mass and is mainly composed of polysaccharides, lipids, proteins and extracellular DNA. Due to its complexity and variability, EPS limits the overall access to bacteria against environmental stress and drug interference also PS penetration. To exert a biofilm clearance effect, it seems reasonable that the PS should pass through the entire matrix and penetrate further to contact the microbial cells. The initial step of penetration into the EPS matrix is influenced by the size, charge and functional groups of PS. The negatively charged EPS matrix is more likely to interact with positively charged PS than negative or neutral PS owing to ionic or hydrophobic interaction. However, the exact interaction between EPS components and various PSs is poorly investigated. Previous studies have demonstrated that cationic PSs could serve as antibacterial agents via interacting with EPS matrix and negatively bacterial membrane. Therefore, some studies have tried to adjust the charge of PS by coating it with nanoparticles to increase the possibility of interaction with microbial cells and further enhance the antimicrobial effect. For instance, Shrestha et al. have conjugated RB with chitosan nanoparticles and found bacteria cells in biofilm increase the uptake of the newly formulated PS (Shrestha et al., 2014). They attributed the increased ability to the positive charge and nano-size of PS when conjugating with chitosan, which resulted in more interaction with bacterial cells and the negatively charged EPS matrix. Another representative example is conjugating the TBO with silver nanoparticles to increase the interaction with bacterial cells and enhance the antimicrobial properties (Misba et al., 2016). The modified PS binds to the EPS matrix via electrostatic interaction and breaks them down, the rest PS molecules could enter the bacterial cell membrane and biofilm structure more easily.

In addition to interacting with the EPS matrix by PS itself, ROS generated exterior to the microbes is also effective at attacking EPS molecules and dispersing the matrix. Mang et al. investigated the efficacy of porfimer sodium as PS against biofilm on different substrates. Porfimer sodium at a final concentration of 40 μM plus 100 J/cm^2^ induced a significant reduction (up to 5 logs), with the dispersion of the matrix according to multiple attenuated internal Reflection – infrared spectroscopy detection (Mang et al., 2016). Later, another research by Wu et al. observed the structure of cariogenic biofilms formed on dentin slices after TBO-mediated aPDT treatment by scanning electron microscopy (Wu et al., 2019). The SEM images revealed that cariogenic biofilms become small sparse colonies rather than a whole tightly connected structure, indicating ROS generated from aPDT process may compromise the EPS matrix and make cells lose interactions. The inhibition of EPS production and destruction of biofilm structure in *S. mutans* biofilm or *E. faecalis* biofilm was also observed in aPDT experiments. Furthermore, some studies were designed to get more comprehensive knowledge of aPDT on specific components of the EPS matrix. Extrapoly saccharide is a huge fraction of the EPS matrix and is indispensable for biofilm formation and maturation. Beirão et al. investigated the effect of cationic porphyrin-mediated PDT on the polysaccharides produced by 24 h-old *P. aeruginosa* biofilms (Beirao et al., 2014). As a result, polysaccharides of biofilms after aPDT treatment decreased to approximately 80% when compared to the control group, suggesting EPS component may act as a photosensitization target. In addition to polysaccharides, eDNA is also an integral part of the biofilm matrix which offers structural integrity to the biofilm and exchanges the genetic information. Garcia et al. found that eDNA content in *C. albicans* was significantly decreased when subjected to TBO-mediated aPDT treatment. The authors claimed that eDNA decrease due to aPDT treatment may be important for reducing antifungal resistance and impairing the structural integrity of *C. albicans* biofilms.

### Morphology destruction

Many electron microscopic techniques and molecular biological methods have been applied to shed light on the mechanisms responsible for ROS damage. With respect to microbial cells in oral biofilms, some literature has demonstrated that aPDT treatment could result in a vague, wrinkled, or broken cell profile in some oral microorganisms (Caminos et al., 2008; Zhao et al., 2018). For instance, Cai et al. have examined the capability of TBO-meditated aPDT on *S. aureus* biofilm formed on titanium disks. According to SEM images, the authors found *S. aureus* cell envelope becomes wrinkled after incubation with TBO and red light. As with the previous finding, Kim et al. have verified the morphology change of *S. mutans* after aPDT treatment (Kim et al., 2021). Their SEM data showed that cell membrane destruction and cytoplasm leakage might be responsible for this loss of biofilm viability. Digo et al. have investigated the details of membrane damage in *E. faecalis* and *C. albicans* biofilms following Zn(II)e6Me-mediated aPDT inactivation (Diogo et al., 2017). Transmission electron microscope (TEM) observation revealed the irreversible damage of *E. faecalis* cells, presenting empty internal structures and intact cell wall, which was called “cell ghost.” As for *C. albicans*, they displayed several changes in the cellular organization, with a cytoplasmic membrane disruption, vacuole formation, and organelles damage, indicating cell autophagy. Atomic force microscopy (AFM) is also an advanced tool for high-resolution imaging to unveil some properties of individual microbial cells after aPDT photoactivation. Severe alteration of an increased surface average roughness in *E. faecalis* cells in the TBO-mediated aPDT-treated groups was found (Lopez-Jimenez et al., 2015). The increase in the cell surface roughness means the disruption of cell wall or membrane and the leakage of cell contents. Apart from microscope observation, flow cytometry in conjunction with some fluorescence could be used to assess the effects of aPDT on cell membrane properties. A representative example is using propidium iodide (PI) dyes to detect cell membrane integrity after aPDT treatment. PI could enter the injured cytoplasmic membrane and bind with DNA emitting red fluorescence. A previous study has found that *E. coli* showed an increase of PI-positive cells after treatment with SAPYR-mediated aPDT, although there were only 2 log_10_ steps in microbial reduction (Muehler et al., 2020).

### Oxidation of cellular components

In addition to the morphology change, the free radicals would attack intercellular components directly or indirectly. Lipids account for the major constituent of the cell membrane and other organelles. It is highlighted that the assessment of lipid peroxidation is an essential biomarker of oxidative stress produced by aPDT procedure. Lipid peroxidation could occur in a contact-dependent way by the collision of excited PSs with targets or in a contact-independent way by the interaction of ^1^O_2_ with unsaturated lipids (Bacellar and Baptista, 2019). Transition metals inside cells, especially Fe liberated from Fe-S clusters, decompose lipid peroxides into free radical and initiate chain reactions related to membrane disruption. Therefore, the consequences of lipid peroxidation are changes in membrane permeability and membrane fluidity, leading to the leakage of ions and metabolites. For example, Meng et al. have demonstrated the high antimicrobial potential of pH-responsive Cur nanoscale zeolitic imidazolate framework 8 in combination with light illumination on gram-positive bacteria and gram-negative bacteria (Meng et al., 2022). The underlying mechanism of the antimicrobial behavior is that aPDT increases the permeability of the bacterial cell membrane with leakages of K^+^, which is assigned to lipid peroxidation due to oxidative stress of aPDT. Another study has assessed the photodynamic oxidation of *E. coli* membrane phospholipids using a lipidomic approach (von Ranke et al., 2018). The findings suggested that phospholipid with more unsaturated fatty acyl chains is prone to oxidation. Besides, the generation of hydroperoxides and hydroxy derivatives was confirmed during the photooxidation of cardiolipins in the presence of a PS. However, research on how PDT attacks biological macromolecules in oral microorganisms is scarce.

Some statements illustrate that the targets of ROS attack are mostly concentrated on the level of the cytoplasmic membrane and cell wall constituents, even though some PS could bind to DNA. However, some results from aPDT studies suggest that DNA damage is one of the crucial mechanisms of cell death. There are several proposed pathways of DNA photocleavage in a review, 1) direct interaction with the excited PS, 2) chemical reactions mediated by ROS, and 3) indirect effect due to secondary intermediates (Epe, 2012). It is demonstrated that aPDT attacks bases and sugar moieties, resulting in the break of single-stranded and double-stranded DNA. By using agarose gel electrophoresis, findings from several studies revealed genomic DNA fragmentation due to photoactivation regardless of the tested microorganisms (Alves et al., 2013; Liu et al., 2015). The photodynamic effect on plasmid DNA has also been investigated by Spesia et al. (2009). Their results showed modifications of plasmid DNA coupling with aggregation of cytoplasmic macromolecules in *E. coil* after long irradiation periods. Irrespective of the pathways, the localization of the PS should be as close as possible to DNA, which is an essential prerequisite for DNA damage. The occurrence of DNA photocleavage is also affected by PS type, concentration and light illumination period. Cationic PS rather than anionic PS could bind to DNA due to the interaction with nucleotide chains via electrostatic contribution (Spesia et al., 2009). The shorter irradiation time is not enough for ROS to be in close proximity with DNA and interact with them, since microbial cells harbor their repairing system to overcome the induced DNA damage (Bertoloni et al., 2000).

### Downregulation of virulence factor

The microorganisms could exert a pathogenic effect and cause infection through multiple virulence factors. The idea of suppressing the virulence factors as a means of controlling biofilm-associated infection has been a promising concept replacing antibiotic use. Previous publications have reported the anti-virulence approaches including inhibition of toxins secretion, block of quorum-sensing communication, and down-regulation of virulence genes (Theuretzbacher and Piddock, 2019). Oxidative stress of aPDT could regulate virulence factors such as biofilm formation, quorum sensing, and antibiotic resistance genes. The most studied examples are that photoactivation leads to the inhibited activity of quorum sensing molecules in *P. aeruginosa* (Tan et al., 2018; Pourhajibagher et al., 2020a).

Although the literature indicates the efficacy of aPDT on oral biofilms, the research about the modulation effect of aPDT on virulence factors of oral microorganisms is scarce. *S. mutans*, as oral gram-positive bacteria, harbors several essential virulence factors to facilitate its cariogenic potential, especially its ability to synthesize water-insoluble polysaccharides promoting adhesion and acidogenicity. GtfB expression in *S. mutans* was dramatically downregulated by irradiating light in combination with various PSs including TBO, ICG and chlorophyllin-phycocyanin (Pourhajibagher et al., 2018a; Afrasiabi et al., 2020). Another study has described the anti-biofilm effects of aPDT mediated by nano-quercetin on *S. mutans*. They concluded the ROS generated by aPDT reduces the metabolic activity and disrupts the biofilm structure by interfering with the quorum sensing system and downregulating the expression of related genes (Pourhajibagher et al., 2022). *Porphyromonas gingivalis* is a periodontal pathogen that is involved in the initiation and maintenance of periodontal inflammation. The fimbriae encoded by *fimA* gene is the most virulence factor of *P. gingivalis*. The presence of fimbriae increases the ability to adhere to salivary protein, collagens, epithelial cells and other bacteria species, thus modulating the biofilm formation and invasion into periodontal tissues. Pourhajibagher et al. have reported the down-regulation effect of aPDT on *fimA* expression, which resulted in the modulation of *P. gingivalis* virulence in surviving cells in the planktonic growth (Pourhajibagher and Bahador, 2017). The regulating effect of aPDT on virulence factor expression of *C. albicans* has also been explored. The genes responsible for biofilm formation and hyphae transition (*ALS3, HWP1, UEG6* and *HGC1*) were suppressed under ALA-mediated aPDT (Shi et al., 2021).

## Combination therapy with aPDT

Throughout the studies we mentioned in this article, we have seen that multi-species and mature biofilms are less susceptible to aPDT treatment. Considering the protection provided by the EPS matrix, it is difficult for PS to penetrate deeply into the biofilm and be absorbed by bacteria. Likewise, the limited diffusion distance and short lifespan of ROS also hinder the lethality of aPDT to pathogens. As aPDT is an essential and promising approach that has its shortcomings, to overcome the limitations, researchers are working on combing aPDT with other substances like antibiotics, antiseptics, and ultrasound techniques to explore a more satisfactory effect in microbial inactivation.

### Antibiotics

It is well established that repeated exposure of bacteria to antibiotics rapidly induces resistance development, which occurs among all classes of antibiotics. To overcome the multi-drug resistant bacterial infection, researchers have attempted to combine the antibiotic with aPDT and expect the combination would act synergistically and lead to a better therapeutic outcome. To date, more and more scientists have investigated the efficiency of different antibiotics/aPDT combinations towards various pathogens (Ronqui et al., 2016; Ucuncu et al., 2020; Zhao et al., 2021). The combined efficacies were assessed with *in vitro* bacterial suspensions/biofilms, *in vivo* models, or (and) in the clinic. Among all studies, only one paper evaluated the association of aPDT with antibiotics for oral biofilms. The authors tested the potential effect of photodithazine (PDZ)-mediated aPDT in combination with local metronidazole application against *F. nucleatum* and *P. gingivalis* biofilms (Tavares et al., 2018). Their experimental outcomes suggested that aPDT seems to make biofilm cells more sensitive to metronidazole penetration and thus lead to a remarkable bactericidal effect. Although many successful cases have been reported, the combination of antibiotics and aPDT does not always guarantee the enhancement of the antibacterial effect. Some studies have reported the unchanged or reduced therapeutic effect of PDT/antibiotic combination therapy compared to the single treatment (Nitzan et al., 1998; Tanaka et al., 2013). The final therapeutic outcome is determined by a series of factors, including the combination of different antibiotics and aPDT, microbial species, and the host environment. For example, Pérez-Laguna et al. investigated the combined effects of gentamicin with MB-mediated aPDT on gram-positive bacteria (*S. aureus*) and gram-negative bacteria (*P. aeruginosa*) (Perez-Laguna et al., 2020). The authors found enhanced levels of killing on two strains in planktonic state when coupling antibiotics and aPDT, however, the combination did not exert a potentiated bactericidal effect against *S. aureus* biofilms. Conversely, the same group reported the desirable killing enhancement on *S. aureus* biofilm following RB-mediated aPDT in combination with gentamicin (Perez-Laguna et al., 2018). Other studies also supported the above conclusion that the combination efficiency depends largely on the complex interaction between antibiotics and aPDT, especially the influence of natural structural properties of the antibiotic and PS. Gram-negative trains are generally less susceptible to the combination treatment versus gram-positive bacteria. This difference is likely due to the profound variation in the cell membrane or cell wall structure between species, which makes their binding efficiency and affinity to antibiotics or PS different.

With respect to the combined mechanisms, some literature has speculated or verified the involved mechanisms according to the observed results. For instance, Ronqui et al. attributed the synergistic antimicrobial effect of aPDT and ciprofloxacin to the ability of aPDT in the disruption of biofilm matrix, turning biofilm cells into planktonic counterparts to become more sensitive to the antibiotics (Ronqui et al., 2016). Another mechanism proposed by Iluz et al., analyzed the synergistic mechanism of aPDT and oxacillin, thinking that ^1^O_2_ produced by aPDT can damage the cell membrane of microorganisms and change the properties of the cell membrane, resulting in increased uptake of antibiotics by microorganisms (Iluz et al., 2018). As the external structure of microorganisms, the cell membrane or cell wall often serves as the first line of defense for microorganisms and is also the target of various antimicrobials. The cell membrane or cell wall of aPDT-treated cells becomes unstable and more permeable, facilitating further interaction with the other antimicrobials.

Findings from the above studies suggested that the simultaneous application of aPDT with antibiotics facilitates the increased killing of microbes, meanwhile, this combination can also reduce the concentration and frequency of antibiotics to avoid side effects like antibiotic resistance. It is clear, however, that further work exploring the potential of this combined application in dentistry is necessary.

### Inorganic salts

A range of non-toxic inorganic salts, such as sodium azide, sodium thiocyanate, and potassium iodide (KI), have been extensively used in association with aPDT to promote its killing of microbes. Among inorganic salts, KI is the most studied, whose potentiated effect has been demonstrated *in vitro* studies and *in vivo* infection models. The addition of KI into aPDT procedure could lead to a significantly improved killing of gram-positive, and gram-negative bacteria, fungi and multi-resistant bacteria (Vecchio et al., 2015; Huang et al., 2017). The potentiation effect on biofilm elimination also has been investigated, Yuan et al. described that KI solution with MB then subsequently irradiated by light, could give rise to the effective killing of an endodontic pathogen (*E. faecalis*) when compared to using PSs alone (Yuan et al., 2019). Their finding further demonstrated that a lower concentration of MB-mediated aPDT plus KI has the same effect as NaClO in eradicating *E. faecalis* biofilm, it was not as toxic to the surrounding cells as NaClO. Moreover, the potentiated antimicrobial effect of KI could avoid the staining effect caused by a high concentration of PS in dentistry applications. Scholars proposed that ROS generated by aPDT procedure could interact with KI to generate iodine species (iodine radicals and free iodine) in two ways (Hamblin, 2017). One is that the triplet state PS would undergo electron transfer to excess iodine ions in solution to form the PS radical cation and the iodine anions. The other is the interaction between ^1^O_2_ and KI to form H_2_O_2_ and molecular iodine. However, the potentiation of antimicrobial killing caused by KI seem to depend strongly on PS type (Vieira et al., 2018). The features of PS like charge distribution, ^1^O_2_ production ability, aggregation behavior, and affinity for the cell membrane should be considered when analyzing the synergistic behavior with KI.

Sodium azide is a classical quencher of ^1^O_2_ and has been used to inhibit the bacteria-killing caused by ^1^O_2_ during aPDT. However, some studies have found interesting results that the addition of sodium azide in some aPDT processes can highly enhance the effectiveness of aPDT in microbial killing, independent of the presence or absence of oxygen (Huang et al., 2012a). Further investigation by the same team shed light on the mechanism that triplet PS could absorb an electron from azide to form azidyl radical, which is more selectively and effectively bactericidal (Yin et al., 2015). Aizdyl radical has a longer lifetime to diffuse into cells and cause damage, though they are as reactive as •OH or ^1^O_2_. The degree of potentiation (or inhibition) of killing due to sodium azide is dependent on various factors, including gram-classification, the binding capacity with bacteria of PS, and PS structure (Kasimova et al., 2014).

### Chemical agent

One of the primary objectives of oral infectious disease is microbial reduction, creating a clean microenvironment that promotes the normal healing process of the surrounding tissues. Chemical agents such as NaClO, H_2_O_2_, CHX, or ethylenediamine tetraacetic Acid (EDTA) are used for irrigants to reduce pathogenic bacteria and control infection. For example, NaClO is often used as an endodontic irrigant owing to its broad antimicrobial activity and ability to dissolve organic tissue. H_2_O_2_ is commonly used as a sterilization agent, disinfectant, and antiseptic in dental practice. EDTA is popularly used as a root canal irrigant to remove smear layers containing bacterial products, dentin debris, and residual pulp tissues. CHX has an antimicrobial effect on bacteria, fungi, and viruses responsible for different oral diseases. It has been demonstrated that CHX could be used as a mouthwash for oral plaque prevention or gel formulations for gingivitis and candidiasis treatment. However, using current antimicrobials alone can not fully eradicate the microorganisms from the focal site, especially in the complex structure of the oral cavity. It is generally accepted that the combined application of two or more antimicrobial treatments could result in more efficient reductions in pathogen amount and consequences of side effects. There is an increasing number of studies exploring the chemical agents with aPDT in combination therapy by taking advantage of the ubiquity and versatility of these chemical agents.

*In vitro* examples of the combination of EDTA with aPDT are focused on caries and root canal infection models. Nima et al. tested the efficacy of Cur-mediated aPDT plus EDTA on *S. mutans* and investigated its involved mechanism (Nima et al., 2021). The combination of Cur-mediated aPDT with EDTA presented a synergistic effect according to the fractional inhibitory concentration index results, and the authors attributed the result to the chelation ability of EDTA, which could alter the permeability of the cell wall and contribute to significant penetration of Cur on the bacteria structure. Another study also used Cur-mediated aPDT plus EDTA to inactive biofilms isolated from infected dentin caries and analyzed the anti-biofilm effectiveness via CLSM (Mendez et al., 2019). Their results were consistent with the later finding that EDTA favors the in-depth action of aPDT by increasing the penetrability of the cellular membrane.

Combining aPDT with H_2_O_2_ has been explored in different investigations, all of which are associated with oral infection models such as pulp infection, periodontitis, peri-implantits, and denture stomatitis. Several studies have demonstrated that the addition of H_2_O_2_ into aPDT procedure exerted a significant antimicrobial enhancement in *E. faceails* biofilm, *S. aureus* biofilm, and even multi-species biofilms (Garcez and Hamblin, 2017; Li et al., 2021; Nie et al., 2021). In a related study by Garcez et al., they discovered that the sequence of H_2_O_2_ application has an impact on final antimicrobial effectiveness, and using H_2_O_2_ followed by MB-mediated aPDT achieves higher disinfection than aPDT alone (Garcez and Hamblin, 2017). Given the promising results, they pointed out that pre-treatment with H_2_O_2_ followed by aPDT appears to be an effective disinfection protocol for endodontic treatment. The authors speculated that H_2_O_2_ might increase the available oxygen concentration to allow more PS to transform into ROS or increase PS penetration into biofilm. Another investigation also emphasized the importance of H_2_O_2_ pretreatment, which could lead to more satisfying results than treating with aPDT at first (Li et al., 2021). Additionally, the authors revealed the mechanism for the enhanced antimicrobial effect due to H_2_O_2_ pretreatment. The verified mechanism is that H_2_O_2_ could degrade EPS of biofilm and increase the outer membrane permeability of microbial cells, thereby facilitating the bacterial uptake of PS.

In addition to EDTA and H_2_O_2_, some studies have attempted to combine aPDT with NaClO or CHX to disinfect oral biofilms more effectively. For example, Cai and coworkers investigated the potentiation of aPDT by 0.2% CHX against *P. gingivals* biofilms *in vitro* (Cai et al., 2019b). Pre-treating biofilm with CHX and then subjected to aPDT had more potential in reducing the viable biofilm counts and destructing the biofilm structure than CHX or aPDT. Though the exact mechanisms of cooperative action of CHX and aPDT have yet to be elucidated in their study, they have postulated several possible modes. Another study analyzed the combination action of CHX and aPDT, they found CHX in association with aPDT significantly downregulates the gene expression of virulence factors determining colonization, adhesion, immunoevasion, and immunosuppression (Bolhari et al., 2018). As acknowledged, a high concentration of NaClO would cause unexpected accidents including mucosa irritation and injury, clothing damage, air emphysema, and allergic reaction. Wang et al. investigated the combination of PDT with different concentrations of NaClO ultrasonic irrigation on a 21-day *in vitro* root infection model (Wang et al., 2015). As a result, they found ultrasonic irrigation with 2.5% NaClO plus MB-mediated PDT could be as comparable as 5.25% NaClO in disinfection and tissue debridement. The results of their experiments concluded that it is feasible to reduce the concentration of NaClO to a safer level while maintaining enough antibacterial efficiency through the synergistic effect of aPDT with NaClO ultrasonic irrigation.

### Ultrasound technique

The efficacy of aPDT in eradicating deeply located infections like root canal infection and periodontitis is hampered by various factors. For instance, the limited penetration depth of PS and light delivery in dentin tubules, as such, microorganisms hidden in the deeper dentin tubule cannot be killed by ROS. The other is deep tissues with a hypoxic microenvironment that is not beneficial for ROS generation.

The ultrasound technique has been developed as an antimicrobial tool when acting alone or in combination with other antimicrobial strategies (LuTheryn et al., 2020). Recently, a promising method of enhancing aPDT efficiency and effectiveness is the combined application of low-frequency ultrasound with aPDT. The biological effects of ultrasound include thermal effect, mechanical effect, cavitational effect, and chemical effect. It is well known that the depth of invasion of microorganisms into dentinal tubules is much deeper than that of PS injection. Some investigations have successfully applied ultrasound techniques to activate PS to increase the penetration depth into the dentinal tubules, resulting in the enhanced killing. Niavarzi et al. used an ultrasonic device to promote the agitation and penetration of MB into the canal and dentin tubules (Niavarzi et al., 2019). Their results suggested that the final antimicrobial activity is greatly enhanced under ultrasonic activation of MB. However, specific PS could be excited by ultrasound and light. As a consequence, the PS is referred to as a sonosensitizer. This new approach uses an ultrasound technique and lights together to activate the PS, which is called sonophotodynamic therapy (SPDT). Alves and colleagues have explored the antifungal activity of aPDT, SDT and SPDT in planktonic *C. albicans* suspension and biofilm (Alves et al., 2018). The study found that, the suspension was eradicated while the biofilm was little influenced following aPDT or SDT application. However, the biofilm biomass and viability were significantly reduced after SPDT treatment by using photodithazine. In another study by the same research group, the authors found that the *S. aureus* reduction induced by Cur-mediated aPDT increase from 2.12 log_10_ to 4.33 log_10_ after being activated with ultrasonic waves (Alves et al., 2021). Although the *in vitro* efficacy has been validated in the published studies, the exact mechanisms underlying the enhancement effect of ultrasound on aPDT lack full investigations. There are several statements accounting for the possible mechanisms of the potentiated effect due to ultrasound. Bao et al. have shown that ultrasound could generate transient pores in the cell membrane of microorganisms (Bao et al., 1997). The changes in cell membrane characteristics enables bacteria to absorb more photosensitizers. In addition, ultrasound waves promote the circulation of the microorganisms in the medium, turning them more directly accessible to light. So far, there are no SPDT treatments on oral microorganisms or biofilm. Also, the SPDT procedure harbors a similar feature to aPDT whose effect is dependent on frequency and energy. Future studies on enlarging the scope of SPDT and optimizing the parameters of SPDT are warranted.

## Conclusion and future perspectives

The oral cavity is a natural habitat for many microbial species responsible for oral infections. For example, dental caries is synergistic activities of acidogenic and aciduric species like streptococcus and lactobacilli species. The accumulation of their secreted byproducts is associated with the local acidification of the oral microenvironment and tooth structure erosion (Valm, 2019). In contrast to caries, the microbiome of endodontic infection depends on communication channel between the endodontic environment and the microbial source. *E. faecalis* is ever highlighted as the main pathogen for endodontic infection. Nevertheless, currently, it is known that other bacteria like *F. nucleatum* and *Propionibacterium* are also dominantly involved in endodontic failure (Prada et al., 2019). As with endodontic infection, the subgingival community orchestrates the initiation and development of periodontitis or peri-implantitis. These microbial communities are mainly gram-negative organisms called the “Red Complex” consisting of *P. gingivalis*, *T. denticola* and *T. forsythia*. These free-floating oral microbial entities live as biofilms called dental plaque, and the relation between dental plaque and oral infectious diseases is clarified. Therefore, it is extremely important to eradicate the notorious biofilm from tooth surface or restorative material surface.

Mechanical debridement, ranging from instrumentation to scaling or root planing, is still considered a common and standard treatment of oral infection based on mechanical disruption. Although it is a conventional option for most oral infectious diseases, it has shortcomings such as incomplete cleaning of anatomical variation, inefficient control of severe infection, time-consuming procedures, and occasionally uncomfortable to the patients. It is generally accepted that some implementation of other adjunctive treatments into oral anti-infective procedures could complement oral mechanical cleaning and result in better clinical outcome (Renvert et al., 2008; Figuero et al., 2014). With the increasing cognition in antibiotic resistance, aPDT has been advocated as a promising and effective approach against oral biofilms. The comparison between aPDT and mechanical cleaning on biofilm elimination has been widely examined and discussed during past decades. Their comparison covers the clearance ability of biofilms, histological changes of oral tissues, and the assessment of the improvement of clinical indicators. The unanimous conclusion drawn is that aPDT cannot replace mechanical cleaning, but can serve as an auxiliary method to improve the treatment effectiveness.

From a clinical perspective, aPDT as an aid to conventional treatment is not a routine therapy for all infection situations. It is essential to discriminate infection stage and degree of oral infection since infection staging can also provide the framework to determine when aPDT needs to be implemented. Take apical periodontitis as an example, conventional chemomechanical instrumentation and medicaments are enough for those conditions with the absence of or minimal changes in the apical tissues. When the apical bone loss extends beyond the immediate apical region, it may indicate the presence of secondary or resistant infection and the need for other interventions like aPDT. As for periodontitis and peri-implantitis, it is suggested that aPDT could be adjunctive use of mechanical debridement for all patients regardless of the disease state. In addition to the timing of the application, different parameters should be optimized to ensure better performance. PS concentration, pre-irradiation time, light emission spectrum required for PS activation, and light dose are essential parameters of aPDT application. The wavelength of irradiated light should match the maximum absorbance of PS. And PS concentration and light dose should reach the antimicrobial threshold without causing heat or injury to surrounding tissues. However, there are no standardized protocols for aPDT against different infections. The clinical effectiveness of aPDT is not solely dependent on PS concentration or light dose but is also influenced by the external microenvironment and biofilm structure. For instance, the oral biofilm could colonize the root canal or deep periodontal pocket where the light is not easily accessible. Besides, these locations are hypoxic that lack enough oxygen to trigger photochemical reactions. A recent study has pointed out sonic and ultrasonic treatments will contribute to the penetration of PS into root canals, which may enhance aPDT efficiency against biofilm formed in deeper dentin tubules (Wang et al., 2023). Another study has revealed that EDTA would destabilize the biofilm matrix of *S. mutans* and enhance aPDT effectiveness on *S.mutans* killing (Nima et al., 2021). These ideas are worthwhile to implement into clinical practice and may contribute to a more successful outcome.

However, some doubts concerning the use of aPDT are needed to be addressed before wide clinical application. Firstly, the toxicity profile of aPDT should be investigated in all aspects, ranging from *in vitro* cytotoxicity experiments to *in vivo* research. Secondly, the concept of maintaining oral innate flora balance must be established during the use of an antimicrobial strategy. However, whether the repeated use of aPDT will lead to oral ecosystem imbalance is a problem that needs to be studied. Thirdly, as the antimicrobial effect of aPDT on multi-species biofilm or complicated infection is limited, it is necessary to combine aPDT with other antibacterial agents. Thus, the compatibility of aPDT with other antimicrobials should be examined to avoid any side effects of cooperative usage. It is worthwhile to investigate the antimicrobial process of aPDT in combination with other antimicrobials more detailly in the future.

Altogether, aPDT has promise for oral anti-biofilm treatments, but they have yet to overcome hurdles for satisfactory clinical outcomes. Moreover, clinicians may also encounter some drawbacks during aPDT treatment. Firstly, due to intrinsic features of the current PSs, such as low quantum yield of ROS and aggregation behavior in aqueous solution, the efficacy of aPDT on complex multi-species biofilm is unsatisfactory. The complex structure like the dentin tubule or hypoxic periodontal pocket also confines the aPDT efficacy. Combining aPDT with other antimicrobials or techniques allows for a more favorable treatment result and reduction of cytotoxicity due to high-concentration use. Nonetheless, studies based on cooperative strategy are still in the primary stage, with a lack of *in vivo* and clinical evidence. In addition, further research is also recommended to shed light on the mechanisms of cooperative strategy. It is worthwhile to investigate the antimicrobial process of aPDT in combination with other antimicrobials more detailly in the future. Secondly, available photosensitizers like MB, TBO, or malachite green are reported to change the color of tooth structure, which further influences teeth esthetics (Costa et al., 2016). The longer the incubation time of PS, the more PS molecules penetrate the internal structure of the tooth. Thirdly, though contact allergy rarely occurs, allergic to photosensitizers or light irradiation is still reported (Wulf and Philipsen, 2004). These drawbacks should be taken into account before the extensive application of aPDT into clinical practice.

## Author contributions

YL and GS contributed to the conception and design of the study and wrote the first draft of the manuscript. JX and SX performed the data searching and wrote sections of the manuscript. YL and CL performed the final corrections. All authors contributed to the article and approved the submitted version.

## Funding

This work was supported by The Youth Grant of the Scientific Research of Fujian Province, China (Grant number: 2019-2-40).

## Conflict of interest

The authors declare that the research was conducted in the absence of any commercial or financial relationships that could be construed as a potential conflict of interest.

## Publisher’s note

All claims expressed in this article are solely those of the authors and do not necessarily represent those of their affiliated organizations, or those of the publisher, the editors and the reviewers. Any product that may be evaluated in this article, or claim that may be made by its manufacturer, is not guaranteed or endorsed by the publisher.

## References

[ref1] Afrasiabi S. Pourhajibagher M. Chiniforush N. Aminian M. Rasi Varaei S. S. Bahador A. (2020). Effects of sub-lethal dose of antimicrobial photodynamic therapy on major virulence traits of *Streptococcus mutans*. Photodiagn. Photodyn. Ther. 32:102044. doi: 10.1016/j.pdpdt.2020.102044, PMID: 33010485

[ref2] Ahmed P. Bukhari I. A. Albaijan R. Sheikh S. A. Vohra F. (2020). The effectiveness of photodynamic and antibiotic gel therapy as an adjunct to mechanical debridement in the treatment of peri-implantitis among diabetic patients. Photodiagn. Photodyn. Ther. 32:102077. doi: 10.1016/j.pdpdt.2020.102077, PMID: 33157330

[ref3] Ahmed A. R. Kamran M. A. Suleman G. Sharif R. A. Alamrey A. A. M. Sulaiman S. A. (2023). Novel use of chloro-aluminum phthalocyanine assisted photodynamic therapy helps in periimplant healing among smoking patients. Photodiagn. Photodyn. Ther. 41:103193. doi: 10.1016/j.pdpdt.2022.103193, PMID: 36343897

[ref4] al Deeb M. Alresayes S. A Mokeem S. Alhenaki A. M. AlHelal A. Vohra F.. (2020). Clinical peri-implant health and biological bone marker levels in tobacco users treated with photodynamic therapy. Photodiagn. Photodyn. Ther. 31:101821. doi: 10.1016/j.pdpdt.2020.101821, PMID: 32422214

[ref5] Al-Ahmad A. Muzafferiy F. Anderson A. C. Wolber J. P. Ratka-Kruger P. Fretwurst T.. (2018). Shift of microbial composition of peri-implantitis-associated oral biofilm as revealed by 16S rRNA gene cloning. J. Med. Microbiol. 67, 332–340. doi: 10.1099/jmm.0.000682, PMID: 29458668

[ref6] ALHarthi S. S. Alamry N. Z. BinShabaib M. S. (2022). Effect of multiple sessions of photodynamic therapy on bone regeneration around dental implants among patients with peri-implantitis. Photodiagn. Photodyn. Ther. 37:102612. doi: 10.1016/j.pdpdt.2021.102612 34740836

[ref7] Ali I. A. A. Levesque C. M. Neelakantan P. (2022). Fsr quorum sensing system modulates the temporal development of *Enterococcus faecalis* biofilm matrix. Mol Oral Microbiol 37, 22–30. doi: 10.1111/omi.12357, PMID: 34862746

[ref8] Almohareb T. Alhamoudi N. Al Deeb M. Bin-Shuwaish M. S. Mokeem S. A. Saad Shafqat S.. (2020). Clinical efficacy of photodynamic therapy as an adjunct to mechanical debridement in the treatment of per-implantitis with abscess. Photodiagn. Photodyn. Ther. 30:101750. doi: 10.1016/j.pdpdt.2020.101750, PMID: 32545150

[ref9] Al-Momani M. M. (2021). Indocyanine-mediated antimicrobial photodynamic therapy promotes superior clinical effects in stage III and grade C chronic periodontitis among controlled and uncontrolled diabetes mellitus: A randomized controlled clinical trial. Photodiagn. Photodyn. Ther. 35:102379. doi: 10.1016/j.pdpdt.2021.102379, PMID: 34087466

[ref10] Alsaif A. Tahmassebi J. F. Wood S. R. (2021). Treatment of dental plaque biofilms using photodynamic therapy: a randomised controlled study. Eur. Arch. Paediatr. Dent. 22, 791–800. doi: 10.1007/s40368-021-00637-y, PMID: 34089515 PMC8526452

[ref11] AlSarhan M. A. Altammami M. A. Alaqeely R. S. AlEbdi A. Jasser R. A. Otaibi D. A.. (2021). Short-term improvement of clinical parameters and microbial diversity in periodontitis patients following Indocyanine green-based antimicrobial photodynamic therapy: A randomized single-blind split-mouth cohort. Photodiagn. Photodyn. Ther. 35:102349. doi: 10.1016/j.pdpdt.2021.102349, PMID: 34033939

[ref12] Alves E. Faustino M. A. Tome J. P. Neves M. G. Tome A. C. Cavaleiro J. A.. (2013). Nucleic acid changes during photodynamic inactivation of bacteria by cationic porphyrins. Bioorg. Med. Chem. 21, 4311–4318. doi: 10.1016/j.bmc.2013.04.065, PMID: 23719285

[ref13] Alves F. Gomes Guimaraes G. Mayumi Inada N. Pratavieira S. Salvador Bagnato V. Kurachi C. (2021). Strategies to improve the antimicrobial efficacy of photodynamic, Sonodynamic, and Sonophotodynamic therapies. Lasers Surg. Med. 53, 1113–1121. doi: 10.1002/lsm.23383, PMID: 33508146

[ref14] Alves F. Pavarina A. C. Mima E. G. O. McHale A. P. Callan J. F. (2018). Antimicrobial sonodynamic and photodynamic therapies against *Candida albicans*. Biofouling 34, 357–367. doi: 10.1080/08927014.2018.1439935, PMID: 29671631

[ref15] Alves-Silva E. G. Arruda-Vasconcelos R. Louzada L. M. de-Jesus-Soares A. Ferraz C. C. R. Almeida J. F. A.. (2023). Effect of antimicrobial photodynamic therapy on the reduction of bacteria and virulence factors in teeth with primary endodontic infection. Photodiagn. Photodyn. Ther. 41:103292. doi: 10.1016/j.pdpdt.2023.103292, PMID: 36681260

[ref16] Andere N. Castro Dos Santos N. C. Araujo C. F. Paz H. E. S. Shaddox L. M. Casarin R. C. V.. (2022). Open flap debridement compared to repeated applications of photodynamic therapy in the treatment of residual pockets: A randomized clinical trial. J. Periodontol. 93, 1671–1681. doi: 10.1002/JPER.22-0059, PMID: 35536044

[ref17] Annunziata M. Donnarumma G. Guida A. Nastri L. Persico G. Fusco A.. (2023). Clinical and microbiological efficacy of indocyanine green-based antimicrobial photodynamic therapy as an adjunct to non-surgical treatment of periodontitis: a randomized controlled clinical trial. Clin. Oral Investig. 27, 2385–2394. doi: 10.1007/s00784-023-04875-w, PMID: 36719506 PMC10159973

[ref18] Apatzidou D. Lappin D. F. Hamilton G. Papadopoulos C. A. Konstantinidis A. Riggio M. P. (2017). Microbiome of peri-implantitis affected and healthy dental sites in patients with a history of chronic periodontitis. Arch. Oral Biol. 83, 145–152. doi: 10.1016/j.archoralbio.2017.07.007, PMID: 28780383

[ref19] Aragao M. G. B. Costa C. Lima R. A. Rodrigues L. K. A. Duarte S. Zanin I. C. J. (2019). Comparative effect of two red lights on *Streptococcus mutans* biofilms and assessment of temperature variances in human teeth during in vitro photodynamic antimicrobial chemotherapy. Photobiomodul. Photomed. Laser Surg. 37, 31–37. doi: 10.1089/photob.2018.4511, PMID: 31050940

[ref20] Araujo N. C. Fontana C. R. Bagnato V. S. Gerbi M. E. (2014). Photodynamic antimicrobial therapy of curcumin in biofilms and carious dentine. Lasers Med. Sci. 29, 629–635. doi: 10.1007/s10103-013-1369-3, PMID: 23793414

[ref21] Armand A. Khani M. Asnaashari M. AliAhmadi A. Shokri B. (2019). Comparison study of root canal disinfection by cold plasma jet and photodynamic therapy. Photodiagn. Photodyn. Ther. 26, 327–333. doi: 10.1016/j.pdpdt.2019.04.023, PMID: 31026615

[ref22] Arweiler N. B. Pietruska M. Pietruski J. Skurska A. Dolinska E. Heumann C.. (2014). Six-month results following treatment of aggressive periodontitis with antimicrobial photodynamic therapy or amoxicillin and metronidazole. Clin. Oral Investig. 18, 2129–2135. doi: 10.1007/s00784-014-1193-6, PMID: 24493231

[ref23] Asnaashari M. Eghbal M. J. Sahba Yaghmayi A. Shokri M. Azari-Marhabi S. (2019). Comparison of antibacterial effects of photodynamic therapy, modified triple antibiotic paste and calcium hydroxide on root canals infected with *Enterococcus faecalis*: an in vitro study. J. Lasers Med. Sci. 10, S23–S29. doi: 10.15171/jlms.2019.S5, PMID: 32021669 PMC6983872

[ref24] Azizi B. Budimir A. Mehmeti B. Jakovljevic S. Bago I. Gjorgievska E.. (2018). Antimicrobial efficacy of photodynamic therapy and light-activated disinfection against bacterial species on titanium dental implants. Int. J. Oral Maxillofac. Implants 33, 831–837. doi: 10.11607/jomi.6423, PMID: 30024999

[ref25] Bacellar I. O. L. Baptista M. S. (2019). Mechanisms of photosensitized lipid oxidation and membrane Permeabilization. ACS Omega 4, 21636–21646. doi: 10.1021/acsomega.9b03244, PMID: 31891041 PMC6933592

[ref26] Balhaddad A. A. AlQranei M. S. Ibrahim M. S. Weir M. D. Martinho F. C. Xu H. H. K.. (2020). Light energy dose and photosensitizer concentration are determinants of effective photo-killing against caries-related biofilms. Int. J. Mol. Sci. 21:7612. doi: 10.3390/ijms21207612, PMID: 33076241 PMC7589159

[ref27] Bao S. Thrall B. D. Miller D. L. (1997). Transfection of a reporter plasmid into cultured cells by sonoporation in vitro. Ultrasound Med. Biol. 23, 953–959. doi: 10.1016/S0301-5629(97)00025-2, PMID: 9300999

[ref28] Barbagallo G. Santagati M. Guni A. Torrisi P. Spitale A. Stefani S.. (2022). Microbiome differences in periodontal, peri-implant, and healthy sites: a cross-sectional pilot study. Clin. Oral Investig. 26, 2771–2781. doi: 10.1007/s00784-021-04253-4, PMID: 34826030

[ref29] Bargrizan M. Fekrazad R. Goudarzi N. Goudarzi N. (2019). Effects of antibacterial photodynamic therapy on salivary mutans streptococci in 5- to 6-year-olds with severe early childhood caries. Lasers Med. Sci. 34, 433–440. doi: 10.1007/s10103-018-2650-2, PMID: 30311085

[ref30] Bassetti M. Schar D. Wicki B. Eick S. Ramseier C. A. Arweiler N. B.. (2014). Anti-infective therapy of peri-implantitis with adjunctive local drug delivery or photodynamic therapy: 12-month outcomes of a randomized controlled clinical trial. Clin. Oral Implants Res. 25, 279–287. doi: 10.1111/clr.12155, PMID: 23560645

[ref31] Batalha V. C. Bueno R. A. Fronchetti Junior E. Mariano J. R. Santin G. C. Freitas K. M. S.. (2021). Dental implants surface in vitro decontamination protocols. Eur. J. Dent. 15, 407–411. doi: 10.1055/s-0040-1721550, PMID: 33285571 PMC8382458

[ref32] Batur Y. B. Erdemir U. Sancakli H. S. (2013). The long-term effect of calcium hydroxide application on dentin fracture strength of endodontically treated teeth. Dent. Traumatol. 29, 461–464. doi: 10.1111/edt.12037, PMID: 23441643

[ref33] Beirao S. Fernandes S. Coelho J. Faustino M. A. Tome J. P. Neves M. G.. (2014). Photodynamic inactivation of bacterial and yeast biofilms with a cationic porphyrin. Photochem. Photobiol. 90, 1387–1396. doi: 10.1111/php.12331, PMID: 25112506

[ref34] Bertoloni G. Lauro F. M. Cortella G. Merchat M. (2000). Photosensitizing activity of hematoporphyrin on *Staphylococcus aureus* cells. Biochim. Biophys. Acta 1475, 169–174. doi: 10.1016/s0304-4165(00)00071-4 10832032

[ref35] Bolhari B. Pourhajibagher M. Bazarjani F. Chiniforush N. Rad M. R. Pirmoazen S.. (2018). Ex vivo assessment of synergic effect of chlorhexidine for enhancing antimicrobial photodynamic therapy efficiency on expression patterns of biofilm-associated genes of *Enterococcus faecalis*. Photodiagn. Photodyn. Ther. doi: 10.1016/j.pdpdt.2018.04.019, PMID: 29709604

[ref36] Brauner A. Fridman O. Gefen O. Balaban N. Q. (2016). Distinguishing between resistance, tolerance and persistence to antibiotic treatment. Nat. Rev. Microbiol. 14, 320–330. doi: 10.1038/nrmicro.2016.34, PMID: 27080241

[ref37] Brookes Z. L. S. Belfield L. A. Ashworth A. Casas-Agustench P. Raja M. Pollard A. J.. (2021). Effects of chlorhexidine mouthwash on the oral microbiome. J. Dent. 113:103768. doi: 10.1016/j.jdent.2021.103768, PMID: 34418463

[ref38] Burgers R. Morsczeck C. Felthaus O. Gosau M. Beck H. C. Reichert T. E. (2019). Correction to: induced surface proteins of *Staphylococcus epidermidis* adhering to titanium implant substrata. Clin. Oral Investig. 23:3139. doi: 10.1007/s00784-019-02879-z, PMID: 30915556

[ref39] Cai Z. Li Y. Wang Y. Chen S. Jiang S. Ge H.. (2019a). Antimicrobial effects of photodynamic therapy with antiseptics on T *Staphylococcus aureus* biofilm on titanium surface. Photodiagn. Photodyn. Ther. 25, 382–388. doi: 10.1016/j.pdpdt.2019.01.024, PMID: 30684671

[ref40] Cai Z. Li Y. Wang Y. Chen S. Jiang S. Ge H.. (2019b). Disinfect *Porphyromonas gingivalis* biofilm on titanium surface with combined application of Chlorhexidine and antimicrobial photodynamic therapy. Photochem. Photobiol. 95, 839–845. doi: 10.1111/php.13060 30481378

[ref41] Camacho-Alonso F. Salinas J. Sanchez-Siles M. Pato-Mourelo J. Cotrina-Veizaga B. D. Ortega N. (2022). Synergistic antimicrobial effect of photodynamic therapy and chitosan on the titanium-adherent biofilms of *Staphylococcus aureus*, *Escherichia coli*, and *Pseudomonas aeruginosa*: an in vitro study. J. Periodontol. 93, e104–e115. doi: 10.1002/JPER.21-0306, PMID: 34541685

[ref42] Caminos D. A. Spesia M. B. Pons P. Durantini E. N. (2008). Mechanisms of *Escherichia coli* photodynamic inactivation by an amphiphilic tricationic porphyrin and 5,10,15,20-tetra(4-N,N,N-trimethylammoniumphenyl) porphyrin. Photochem. Photobiol. Sci. 7, 1071–1078. doi: 10.1039/b804965c, PMID: 18754054

[ref43] Cieplik F. Spath A. Leibl C. Gollmer A. Regensburger J. Tabenski L.. (2014). Blue light kills *Aggregatibacter actinomycetemcomitans* due to its endogenous photosensitizers. Clin. Oral Investig. 18, 1763–1769. doi: 10.1007/s00784-013-1151-8, PMID: 24297656

[ref44] Cieplik F. Steinwachs V.-S. Muehler D. Hiller K.-A. Thurnheer T. Belibasakis G. N.. (2018). Phenalen-1-one-mediated antimicrobial photodynamic therapy: antimicrobial efficacy in a periodontal biofilm model and flow Cytometric evaluation of cytoplasmic membrane damage. Front. Microbiol. 9:688. doi: 10.3389/fmicb.2018.00688 29681899 PMC5897782

[ref45] Costa F. O. Esteves Lima R. P. Costa A. M. Costa A. A. Mattos Pereira G. H. Cortelli S. C.. (2023). Adjunctive effects of photodynamic therapy using indocyanine green in residual pockets during periodontal maintenance therapy: A split-mouth randomized controlled trial. J. Periodontol. doi: 10.1002/JPER.22-0672, PMID: 37051740

[ref46] Costa L. M. Matos F. S. Correia A. M. O. Carvalho N. C. Faria-e-Silva A. L. Paranhos L. R.. (2016). Tooth color change caused by photosensitizers after photodynamic therapy: an in vitro study. J. Photochem. Photobiol. B 160, 225–228. doi: 10.1016/j.jphotobiol.2016.04.019, PMID: 27115871

[ref47] Costa-Santos L. Silva-Junior Z. S. Sfalcin R. A. Mota A. Horliana A. Motta L. J.. (2019). The effect of antimicrobial photodynamic therapy on infected dentin in primary teeth: A randomized controlled clinical trial protocol. Medicine (Baltimore) 98:e15110. doi: 10.1097/MD.0000000000015110, PMID: 30985667 PMC6485871

[ref48] Couto A. C. F. de Carvalho R. V. H. Brancini G. T. P. Martins F. G. Sorgi C. A. da Silva R. A. B.. (2021). Photosensitizers attenuate LPS-induced inflammation: implications in dentistry and general health. Lasers Med. Sci. 36, 913–926. doi: 10.1007/s10103-020-03180-2, PMID: 33150475

[ref49] Cugini C. Shanmugam M. Landge N. Ramasubbu N. (2019). The role of exopolysaccharides in Oral biofilms. J. Dent. Res. 98, 739–745. doi: 10.1177/0022034519845001, PMID: 31009580 PMC6589894

[ref50] Cusicanqui Méndez D. A. Gutierres E. José Dionisio E. Afonso Rabelo Buzalaf M. Cardoso Oliveira R. Andrade Moreira Machado M. A.. (2018). Curcumin-mediated antimicrobial photodynamic therapy reduces the viability and vitality of infected dentin caries microcosms. Photodiagn. Photodyn. Ther. 24, 102–108. doi: 10.1016/j.pdpdt.2018.09.007, PMID: 30240927

[ref51] Darmani H. Tawalbeh K. H. Al-Hiyasat A. S. Al-Akhras M. A. (2018). Comparison of the photosensitivity of biofilms of different genera of cariogenic Bacteria in tooth slices. Pol. J. Microbiol. 67, 455–462. doi: 10.21307/pjm-2018-053, PMID: 30550231 PMC7256787

[ref52] De Furio M. Ahn S. J. Burne R. A. Hagen S. J. (2017). Oxidative stressors modify the response of *Streptococcus mutans* to its competence signal peptides. Appl. Environ. Microbiol. 83:e01345-17. doi: 10.1128/AEM.01345-17, PMID: 28887419 PMC5666127

[ref53] de Melo Soares M. S. D'Almeida Borges C. de Mendonca Invernici M. Frantz F. G. de Figueiredo L. C. de Souza S. L. S.. (2019). Antimicrobial photodynamic therapy as adjunct to non-surgical periodontal treatment in smokers: a randomized clinical trial. Clin. Oral Investig. 23, 3173–3182. doi: 10.1007/s00784-018-2740-3, PMID: 30392080

[ref54] de Oliveira R. R. Schwartz-Filho H. O. Novaes A. B. Jr. Taba M. Jr. (2007). Antimicrobial photodynamic therapy in the non-surgical treatment of aggressive periodontitis: a preliminary randomized controlled clinical study. J. Periodontol. 78, 965–973. doi: 10.1902/jop.2007.060494, PMID: 17539707

[ref55] de Sousa Farias S. S. Nemezio M. A. Corona S. A. Aires C. P. Borsatto M. C. (2016). Effects of low-level laser therapy combined with toluidine blue on polysaccharides and biofilm of *Streptococcus mutans*. Lasers Med. Sci. 31, 1011–1016. doi: 10.1007/s10103-016-1944-5, PMID: 27147073

[ref56] Deeb M. A. Alsahhaf A. Mubaraki S. A. Alhamoudi N. Al-Aali K. A. Abduljabbar T. (2020). Clinical and microbiological outcomes of photodynamic and systemic antimicrobial therapy in smokers with peri-implant inflammation. Photodiagn. Photodyn. Ther. 29:101587. doi: 10.1016/j.pdpdt.2019.101587 31689510

[ref57] Del Carpio-Perochena A. E. Bramante C. M. Duarte M. A. Cavenago B. C. Villas-Boas M. H. Graeff M. S.. (2011). Biofilm dissolution and cleaning ability of different irrigant solutions on intraorally infected dentin. J. Endod. 37, 1134–1138. doi: 10.1016/j.joen.2011.04.013, PMID: 21763908

[ref58] Ding C. Zhang F. Gao Y. Li Y. Cheng D. Wang J.. (2021). Antibacterial photodynamic treatment of *Porphyromonas gingivalis* with toluidine blue O and a NonLaser red light source enhanced by Dihydroartemisinin. Photochem. Photobiol. 97, 377–384. doi: 10.1111/php.13333, PMID: 32959424

[ref59] Diogo P. Fernandes C. Caramelo F. Mota M. Miranda I. M. Faustino M. A. F.. (2017). Antimicrobial photodynamic therapy against endodontic *enterococcus faecalis* and *Candida albicans* mono and mixed biofilms in the presence of photosensitizers: A comparative study with classical endodontic Irrigants. Front. Microbiol. 8:498. doi: 10.3389/fmicb.2017.00498, PMID: 28424663 PMC5371592

[ref60] Dobson J. Wilson M. (1992). Sensitization of oral bacteria in biofilms to killing by light from a low-power laser. Arch. Oral Biol. 37, 883–887. doi: 10.1016/0003-9969(92)90058-G, PMID: 1334649

[ref61] Dragana R. Jelena M. Jovan M. Biljana N. Dejan M. (2023). Antibacterial efficiency of adjuvant photodynamic therapy and high-power diode laser in the treatment of young permanent teeth with chronic periapical periodontitis. A prospective clinical study. Photodiagn. Photodyn. Ther. 41:103129. doi: 10.1016/j.pdpdt.2022.103129, PMID: 36156313

[ref62] Eick S. Meier I. Spoerle F. Bender P. Aoki A. Izumi Y.. (2017). In vitro-activity of Er:YAG laser in comparison with other treatment modalities on biofilm ablation from implant and tooth surfaces. PLoS One 12:e0171086. doi: 10.1371/journal.pone.0171086, PMID: 28125700 PMC5268770

[ref63] Elsadek M. F. Almoajel A. Sonbol A. M. Aljarbou H. M. (2023). Chloro-aluminum phthalocyanine-mediated photodynamic therapy improves peri-implant parameters and crevicular fluid cytokine levels in cigarette smokers with chronic hyperglycemia. Photodiagn. Photodyn. Ther. 41:103309. doi: 10.1016/j.pdpdt.2023.103309, PMID: 36709015

[ref64] Epe B. (2012). DNA damage spectra induced by photosensitization. Photochem. Photobiol. Sci. 11, 98–106. doi: 10.1039/c1pp05190c, PMID: 21901212

[ref65] Etemadi A. Eftekhari Bayati S. Pourhajibagher M. Chiniforush N. (2020). In vitro effect of antimicrobial photodynamic therapy with phycocyanin on *Aggregatibacter actinomycetemcomitans* biofilm on SLA titanium discs. Photodiagn. Photodyn. Ther. 32:102062. doi: 10.1016/j.pdpdt.2020.102062, PMID: 33068819

[ref66] Feuerstein O. Persman N. Weiss E. I. (2004). Phototoxic effect of visible light on *Porphyromonas gingivalis* and *Fusobacterium nucleatum*: an in vitro study. Photochem. Photobiol. 80, 412–415. doi: 10.1562/0031-8655(2004)080<0412:PEOVLO>2.0.CO;2, PMID: 15623322

[ref67] Figuero E. Graziani F. Sanz I. Herrera D. Sanz M. (2014). Management of peri-implant mucositis and peri-implantitis. Periodontol. 66, 255–273. doi: 10.1111/prd.12049, PMID: 25123773

[ref68] Flemming H.-C. Wingender J. (2010). The biofilm matrix. Nat. Rev. Microbiol. 8, 623–633. doi: 10.1038/nrmicro2415, PMID: 20676145

[ref69] Garcez A. S. Arantes-Neto J. G. Sellera D. P. Fregnani E. R. (2015). Effects of antimicrobial photodynamic therapy and surgical endodontic treatment on the bacterial load reduction and periapical lesion healing. Three years follow up. Photodiagn. Photodyn. Ther. 12, 575–580. doi: 10.1016/j.pdpdt.2015.06.002, PMID: 26071387

[ref70] Garcez A. S. Hamblin M. R. (2017). Methylene blue and hydrogen peroxide for photodynamic inactivation in root canal - A new protocol for use in Endodontics. Eur. Endod. J. 2. doi: 10.5152/eej.2017.17023, PMID: 29528043 PMC5844508

[ref71] Garcez A. S. Nunez S. C. Hamblim M. R. Suzuki H. Ribeiro M. S. (2010). Photodynamic therapy associated with conventional endodontic treatment in patients with antibiotic-resistant microflora: a preliminary report. J. Endod. 36, 1463–1466. doi: 10.1016/j.joen.2010.06.001, PMID: 20728710

[ref72] Garcia de Carvalho G. Sanchez-Puetate J. C. Donatoni M. C. Maquera Huacho P. M. de Souza Rastelli A. N. de Oliveira K. T.. (2020). Photodynamic inactivation using a chlorin-based photosensitizer with blue or red-light irradiation against single-species biofilms related to periodontitis. Photodiagn. Photodyn. Ther. 31:101916. doi: 10.1016/j.pdpdt.2020.101916, PMID: 32645434

[ref73] Garcia M. T. Ward R. Goncalves N. M. F. Pedroso L. L. C. Neto J. Strixino J. F.. (2021). Susceptibility of dental caries microcosm biofilms to photodynamic therapy mediated by Fotoenticine. Pharmaceutics 13. doi: 10.3390/pharmaceutics13111907, PMID: 34834321 PMC8619263

[ref74] Ghasemi M. Etemadi A. Nedaei M. Chiniforush N. Pourhajibagher M. (2019). Antimicrobial efficacy of photodynamic therapy using two different light sources on the titanium-adherent biofilms of *Aggregatibacter actinomycetemcomitans*: an in vitro study. Photodiagn. Photodyn. Ther. 26, 85–89. doi: 10.1016/j.pdpdt.2019.03.004, PMID: 30836212

[ref75] Gomes-Filho J. E. Sivieri-Araujo G. Sipert C. R. da Silva Santos L. M. de Azevedo Queiroz I. O. Men Martins C.. (2016). Evaluation of photodynamic therapy on fibroblast viability and cytokine production. Photodiagn. Photodyn. Ther. 13, 97–100. doi: 10.1016/j.pdpdt.2016.01.007, PMID: 26796031

[ref76] Grzech-Lesniak K. Gaspirc B. Sculean A. (2019). Clinical and microbiological effects of multiple applications of antibacterial photodynamic therapy in periodontal maintenance patients. A randomized controlled clinical study. Photodiagn. Photodyn. Ther. 27, 44–50. doi: 10.1016/j.pdpdt.2019.05.028, PMID: 31125767

[ref77] Guimaraes L. D. S. da Silva E. A. B. Hespanhol F. G. Fontes K. Antunes L. A. A. Antunes L. S. (2021). Effect of photobiomodulation on post-operative symptoms in teeth with asymptomatic apical periodontitis treated with foraminal enlargement: A randomized clinical trial. Int. Endod. J. 54, 1708–1719. doi: 10.1111/iej.13593, PMID: 34173988

[ref78] Hamblin M. R. (2017). Potentiation of antimicrobial photodynamic inactivation by inorganic salts. Expert Rev. Anti-Infect. Ther. 15, 1059–1069. doi: 10.1080/14787210.2017.1397512, PMID: 29084463 PMC5706449

[ref79] Hoedke D. Enseleit C. Gruner D. Dommisch H. Schlafer S. Dige I.. (2017). Effect of photodynamic therapy in combination with various irrigation protocols on an endodontic multispecies biofilm ex vivo. Int. Endod. J. 51, e23–e34. doi: 10.1111/iej.12763 28276583

[ref80] Hosseinpour-Nader A. Karimi N. Ghafari H. A. Ghorbanzadeh R. (2022). Effect of nanomicelle curcumin-based photodynamic therapy on the dynamics of white spot lesions and virulence of *Streptococcus mutans* in patients undergoing fixed orthodontic treatment: A randomized double-blind clinical trial. Photodiagn. Photodyn. Ther. 40:103183. doi: 10.1016/j.pdpdt.2022.103183, PMID: 36602066

[ref81] Huang Y. Chen Y. Zhang L. H. (2020). The roles of microbial cell-cell chemical communication Systems in the Modulation of antimicrobial resistance. Antibiotics (Basel) 9:779. doi: 10.3390/antibiotics9110779, PMID: 33171916 PMC7694446

[ref82] Huang L. St Denis T. G. Xuan Y. Huang Y. Y. Tanaka M. Zadlo A.. (2012a). Paradoxical potentiation of methylene blue-mediated antimicrobial photodynamic inactivation by sodium azide: role of ambient oxygen and azide radicals. Free Radic. Biol. Med. 53, 2062–2071. doi: 10.1016/j.freeradbiomed.2012.09.006 23044264 PMC3522421

[ref83] Huang L. Szewczyk G. Sarna T. Hamblin M. R. (2017). Potassium iodide potentiates broad-Spectrum antimicrobial photodynamic inactivation using Photofrin. ACS Infect. Dis. 3, 320–328. doi: 10.1021/acsinfecdis.7b00004, PMID: 28207234 PMC5528003

[ref84] Huang L. Xuan Y. Koide Y. Zhiyentayev T. Tanaka M. Hamblin M. R. (2012b). Type I and type II mechanisms of antimicrobial photodynamic therapy: an in vitro study on gram-negative and gram-positive bacteria. Lasers Surg. Med. 44, 490–499. doi: 10.1002/lsm.22045 22760848 PMC3428129

[ref85] Iluz N. Maor Y. Keller N. Malik Z. (2018). The synergistic effect of PDT and oxacillin on clinical isolates of *Staphylococcus aureus*. Lasers Surg. Med. 50, 535–551. doi: 10.1002/lsm.22785, PMID: 29333608

[ref86] Javed F. BinShabaib M. S. Alharthi S. S. Qadri T. (2017). Role of mechanical curettage with and without adjunct antimicrobial photodynamic therapy in the treatment of peri-implant mucositis in cigarette smokers: A randomized controlled clinical trial. Photodiagn. Photodyn. Ther. 18, 331–334. doi: 10.1016/j.pdpdt.2017.04.015, PMID: 28457847

[ref87] Karygianni L. Ruf S. Follo M. Hellwig E. Bucher M. Anderson A. C.. (2014). Novel broad-Spectrum antimicrobial Photoinactivation of in situ Oral biofilms by visible light plus water-filtered infrared A. Appl. Environ. Microbiol. 80, 7324–7336. doi: 10.1128/AEM.02490-14, PMID: 25239897 PMC4249165

[ref88] Kasimova K. R. Sadasivam M. Landi G. Sarna T. Hamblin M. R. (2014). Potentiation of photoinactivation of gram-positive and gram-negative bacteria mediated by six phenothiazinium dyes by addition of azide ion. Photochem. Photobiol. Sci. 13, 1541–1548. doi: 10.1039/c4pp00021h, PMID: 25177833 PMC4208731

[ref89] Kassa C. T. Salviatto L. T. C. Tortamano A. Rost-Lima K. S. Damante C. A. Pavani C.. (2023). Antimicrobial photodynamic therapy mediated by methylene blue in surfactant vehicle as adjuvant to periodontal treatment. Randomized, controlled, double-blind clinical trial. Photodiagn. Photodyn. Ther. 41:103194. doi: 10.1016/j.pdpdt.2022.103194, PMID: 36402375

[ref90] Katalinic I. Budimir A. Bosnjak Z. Jakovljevic S. Anic I. (2019). The photo-activated and photo-thermal effect of the 445/970 nm diode laser on the mixed biofilm inside root canals of human teeth in vitro: A pilot study. Photodiagn. Photodyn. Ther. 26, 277–283. doi: 10.1016/j.pdpdt.2019.04.014, PMID: 30995521

[ref91] Kim M. M. Darafsheh A. (2020). Light sources and Dosimetry techniques for photodynamic therapy. Photochem. Photobiol. 96, 280–294. doi: 10.1111/php.13219, PMID: 32003006

[ref92] Kim J. Kim S. Lee K. Kim R. H. Hwang K. T. (2021). Antibacterial photodynamic inactivation of Fagopyrin F from Tartary buckwheat (*Fagopyrum tataricum*) flower against *Streptococcus mutans* and its biofilm. Int. J. Mol. Sci. 22:6205. doi: 10.3390/ijms22126205, PMID: 34201389 PMC8226997

[ref93] Kim D. Liu Y. Benhamou R. I. Sanchez H. Simón-Soro Á. Li Y.. (2018). Bacterial-derived exopolysaccharides enhance antifungal drug tolerance in a cross-kingdom oral biofilm. ISME J. 12, 1427–1442. doi: 10.1038/s41396-018-0113-1, PMID: 29670217 PMC5955968

[ref94] Kim Y. Park H. Lee J. Seo H. Lee S. (2020). Effect of Indocyanine green and infrared diode laser to *Streptococcus mutans* biofilms. Photobiomodul. Photomed. Laser Surg. 38, 646–652. doi: 10.1089/photob.2019.4796, PMID: 32758054

[ref95] Kohanski M. A. Dwyer D. J. Hayete B. Lawrence C. A. Collins J. J. (2007). A common mechanism of cellular death induced by bactericidal antibiotics. Cells 130, 797–810. doi: 10.1016/j.cell.2007.06.049, PMID: 17803904

[ref96] Kotsakis G. A. Olmedo D. G. (2021). Peri-implantitis is not periodontitis: scientific discoveries shed light on microbiome-biomaterial interactions that may determine disease phenotype. Periodontol. 86, 231–240. doi: 10.1111/prd.12372 33690947

[ref97] Kuga M. C. Gouveia-Jorge E. Tanomaru-Filho M. Guerreiro-Tanomaru J. M. Bonetti-Filho I. Faria G. (2011). Penetration into dentin of sodium hypochlorite associated with acid solutions. Oral Surg. Oral Med. Oral Pathol. Oral Radiol. Endod. 112, e155–e159. doi: 10.1016/j.tripleo.2011.05.040, PMID: 22018593

[ref98] Kwack K. H. Jang E. Y. Yang S. B. Lee J. H. Moon J. H. (2022). Genomic and phenotypic comparison of *Prevotella intermedia* strains possessing different virulence in vivo. Virulence 13, 1133–1145. doi: 10.1080/21505594.2022.2095718, PMID: 35791444 PMC9262359

[ref99] Labban N. Shibani N. A. Al-Kattan R. Alfouzan A. F. Binrayes A. Assery M. K. (2021). Clinical, bacterial, and inflammatory outcomes of indocyanine green-mediated photodynamic therapy for treating periimplantitis among diabetic patients: A randomized controlled clinical trial. Photodiagn. Photodyn. Ther. 35:102350. doi: 10.1016/j.pdpdt.2021.102350, PMID: 34033934

[ref100] Lacerda Rangel Esper M. Â. Junqueira J. C. Uchoa A. F. Bresciani E. Nara de Souza Rastelli A. Navarro R. S.. (2019). Photodynamic inactivation of planktonic cultures and *Streptococcus mutans* biofilms for prevention of white spot lesions during orthodontic treatment: an in vitro investigation. Am. J. Orthod. Dentofac. Orthop. 155, 243–253. doi: 10.1016/j.ajodo.2018.03.027, PMID: 30712696

[ref101] Lamont R. J. Koo H. Hajishengallis G. (2018). The oral microbiota: dynamic communities and host interactions. Nat. Rev. Microbiol. 16, 745–759. doi: 10.1038/s41579-018-0089-x, PMID: 30301974 PMC6278837

[ref102] Leal C. R. L. Alvarenga L. H. Oliveira-Silva T. Kato I. T. Godoy-Miranda B. Bussadori S. K.. (2017). Antimicrobial photodynamic therapy on *Streptococcus mutans* is altered by glucose in the presence of methylene blue and red LED. Photodiagn. Photodyn. Ther. 19, 1–4. doi: 10.1016/j.pdpdt.2017.04.004, PMID: 28414082

[ref103] Leelanarathiwat K. Katsuta Y. Katsuragi H. Watanabe F. (2020). Antibacterial activity of blue high-power light-emitting diode-activated flavin mononucleotide against *Staphylococcus aureus* biofilm on a sandblasted and etched surface. Photodiagn. Photodyn. Ther. 31:101855. doi: 10.1016/j.pdpdt.2020.101855, PMID: 32512247

[ref104] Li Y. Du J. Huang S. Wang S. Wang Y. Cai Z.. (2021). Hydrogen peroxide potentiates antimicrobial photodynamic therapy in eliminating *Candida albicans* and *Streptococcus mutans* dual-species biofilm from denture base. Photodiagn. Photodyn. Ther. 37:102691. doi: 10.1016/j.pdpdt.2021.102691, PMID: 34921987

[ref105] Li X. Liu Y. Yang X. Li C. Song Z. (2022). The Oral microbiota: community composition, influencing factors, pathogenesis, and interventions. Front. Microbiol. 13:895537. doi: 10.3389/fmicb.2022.895537, PMID: 35572634 PMC9100676

[ref106] Lin H. Chen D. Wang M. Lin J. Li B. Xie S. (2011). Influence of pulse-height discrimination threshold for photon counting on the accuracy of singlet oxygen luminescence measurement. J. Opt. 13:125301. doi: 10.1088/2040-8978/13/12/125301

[ref107] Lin Y. Chen J. Zhou X. Li Y. (2021). Inhibition of *Streptococcus mutans* biofilm formation by strategies targeting the metabolism of exopolysaccharides. Crit. Rev. Microbiol. 47, 667–677. doi: 10.1080/1040841X.2021.1915959, PMID: 33938347

[ref108] Liu C. Zhou Y. Wang L. Han L. Lei J. Ishaq H. M.. (2015). Mechanistic aspects of the photodynamic inactivation of vancomycin-resistant enterococci mediated by 5-aminolevulinic acid and 5-aminolevulinic acid methyl ester. Curr. Microbiol. 70, 528–535. doi: 10.1007/s00284-014-0757-7, PMID: 25502688

[ref109] Lopez-Jimenez L. Fuste E. Martinez-Garriga B. Arnabat-Dominguez J. Vinuesa T. Vinas M. (2015). Effects of photodynamic therapy on *Enterococcus faecalis* biofilms. Lasers Med. Sci. 30, 1519–1526. doi: 10.1007/s10103-015-1749-y, PMID: 25917515 PMC4475243

[ref110] LuTheryn G. Glynne-Jones P. Webb J. S. Carugo D. (2020). Ultrasound-mediated therapies for the treatment of biofilms in chronic wounds: a review of present knowledge. Microb. Biotechnol. 13, 613–628. doi: 10.1111/1751-7915.13471, PMID: 32237219 PMC7111087

[ref111] Mang T. Rogers S. Keinan D. Honma K. Baier R. (2016). Antimicrobial photodynamic therapy (aPDT) induction of biofilm matrix architectural and bioadhesive modifications. Photodiagn. Photodyn. Ther. 13, 22–28. doi: 10.1016/j.pdpdt.2015.11.007, PMID: 26617192

[ref112] Martins L. F. B. de Sena L. R. de Paula D. M. Feitosa V. P. Horliana A. Fernandes K. P. S.. (2023). Investigation on the effect of antimicrobial photodynamic therapy as an adjunct for management of deep caries lesions-study protocol for a randomized, parallel groups, controlled clinical trial. Trials 24:165. doi: 10.1186/s13063-023-07181-8 36870982 PMC9985277

[ref113] Matsushima Y. Yashima A. Fukaya M. Shirakawa S. Ohshima T. Kawai T.. (2021). Effects of antimicrobial photodynamic therapy on organic solution and root surface in vitro. Antibiotics (Basel) 10:101. doi: 10.3390/antibiotics10020101, PMID: 33494221 PMC7909815

[ref114] Melo M. A. Rolim J. P. Passos V. F. Lima R. A. Zanin I. C. Codes B. M.. (2015). Photodynamic antimicrobial chemotherapy and ultraconservative caries removal linked for management of deep caries lesions. Photodiagn. Photodyn. Ther. 12, 581–586. doi: 10.1016/j.pdpdt.2015.09.005, PMID: 26431977

[ref115] Mendez D. A. C. Gutierrez E. Dionisio E. J. Oliveira T. M. Buzalaf M. A. R. Rios D.. (2018). Effect of methylene blue-mediated antimicrobial photodynamic therapy on dentin caries microcosms. Lasers Med. Sci. 33, 479–487. doi: 10.1007/s10103-017-2379-3, PMID: 29119417

[ref116] Mendez D. A. C. Rizzato V. L. Lamarque G. C. C. Dionisio E. J. Buzalaf M. A. R. Rios D.. (2019). Could a chelant improve the effect of curcumin-mediated photodynamic antimicrobial chemotherapy against dental intact biofilms? Lasers Med. Sci. 34, 1185–1192. doi: 10.1007/s10103-018-02708-x, PMID: 30604346

[ref117] Meng X. Guan J. Lai S. Fang L. Su J. (2022). pH-responsive curcumin-based nanoscale ZIF-8 combining chemophotodynamic therapy for excellent antibacterial activity. RSC Adv. 12, 10005–10013. doi: 10.1039/D1RA09450E, PMID: 35424930 PMC8966386

[ref118] Misba L. Kulshrestha S. Khan A. U. (2016). Antibiofilm action of a toluidine blue O-silver nanoparticle conjugate on *Streptococcus mutans*: a mechanism of type I photodynamic therapy. Biofouling 32, 313–328. doi: 10.1080/08927014.2016.1141899, PMID: 26905507

[ref119] Mombelli A. Müller N. Cionca N. (2012). The epidemiology of peri-implantitis. Clin. Oral Implants Res. 23, 67–76. doi: 10.1111/j.1600-0501.2012.02541.x 23062130

[ref120] Moore C. Wallis C. Melnick J. L. Kuns M. D. (1972). Photodynamic treatment of herpes keratitis. Infect. Immun. 5, 169–171. doi: 10.1128/iai.5.2.169-171.1972, PMID: 4635496 PMC422342

[ref121] Moradi M. Fazlyab M. Pourhajibagher M. Chiniforush N. (2021). Antimicrobial action of photodynamic therapy on *Enterococcus faecalis* biofilm using curing light, curcumin and riboflavin. Aust. Endod. J. 48, 274–282. doi: 10.1111/aej.12565, PMID: 34529329

[ref122] Moreira S. A. Nunes J. B. Colombo F. A. Fonseca N. Viola N. V. (2021). Radiographic and antimicrobial evaluation of *enterococcus Faecalis* and *Actinomyces Israelii* micro-organisms after photodynamic therapy (aPDT). Photodiagn. Photodyn. Ther. 35:102433. doi: 10.1016/j.pdpdt.2021.102433, PMID: 34256171

[ref123] Muehler D. Rupp C. M. Keceli S. Brochhausen C. Siegmund H. Maisch T.. (2020). Insights into mechanisms of antimicrobial photodynamic action toward biofilms using Phenalen-1-one derivatives as photosensitizers. Front. Microbiol. 11:589364. doi: 10.3389/fmicb.2020.589364, PMID: 33193252 PMC7662152

[ref124] Niavarzi S. Pourhajibagher M. Khedmat S. Ghabraei S. Chiniforush N. Bahador A. (2019). Effect of ultrasonic activation on the efficacy of antimicrobial photodynamic therapy: evaluation of penetration depth of photosensitizer and elimination of *Enterococcus faecalis* biofilms. Photodiagn. Photodyn. Ther. 27, 362–366. doi: 10.1016/j.pdpdt.2019.06.001, PMID: 31176763

[ref125] Niazi F. H. Noushad M. Tanvir S. B. Ali S. Al-Khalifa K. S. Qamar Z.. (2020). Antimicrobial efficacy of indocyanine green-mediated photodynamic therapy compared with Salvadora persica gel application in the treatment of moderate and deep pockets in periodontitis. Photodiagn. Photodyn. Ther. 29:101665. doi: 10.1016/j.pdpdt.2020.101665, PMID: 31978565

[ref126] Nie M. Deng D. M. Wu Y. de Oliveira K. T. Bagnato V. S. Crielaard W.. (2020). Photodynamic inactivation mediated by methylene blue or chlorin e6 against *Streptococcus mutans* biofilm. Photodiagn. Photodyn. Ther. 31:101817. doi: 10.1016/j.pdpdt.2020.101817, PMID: 32407890

[ref127] Nie M. Silva R. C. E. de Oliveira K. T. Bagnato V. S. de Souza Rastelli A. N. Crielaard W.. (2021). Synergetic antimicrobial effect of chlorin e6 and hydrogen peroxide on multi-species biofilms. Biofouling 37, 656–665. doi: 10.1080/08927014.2021.1954169, PMID: 34304642

[ref128] Nikinmaa S. Alapulli H. Auvinen P. Vaara M. Rantala J. Kankuri E.. (2020). Dual-light photodynamic therapy administered daily provides a sustained antibacterial effect on biofilm and prevents *Streptococcus mutans* adaptation. PLoS One 15:e0232775. doi: 10.1371/journal.pone.0232775, PMID: 32374766 PMC7202659

[ref129] Nima G. Soto-Montero J. Alves L. A. Mattos-Graner R. O. Giannini M. (2021). Photodynamic inactivation of *Streptococcus mutans* by curcumin in combination with EDTA. Dent. Mater. 37, e1–e14. doi: 10.1016/j.dental.2020.09.015, PMID: 33143940

[ref130] Nitzan Y. Balzam-Sudakevitz A. Ashkenazi H. (1998). Eradication of *Acinetobacter baumannii* by photosensitized agents in vitro. J. Photochem. Photobiol. B 42, 211–218. doi: 10.1016/s1011-1344(98)00073-6 9595710

[ref131] Okamoto C. B. Bussadori S. K. Prates R. A. da Mota A. C. C. Tempestini Horliana A. C. R. Fernandes K. P. S.. (2020). Photodynamic therapy for endodontic treatment of primary teeth: A randomized controlled clinical trial. Photodiagn. Photodyn. Ther. 30:101732. doi: 10.1016/j.pdpdt.2020.101732, PMID: 32171875

[ref132] Perez-Laguna V. Garcia-Luque I. Ballesta S. Perez-Artiaga L. Lampaya-Perez V. Rezusta A.. (2020). Photodynamic therapy using methylene blue, combined or not with gentamicin, against *Staphylococcus aureus* and *Pseudomonas aeruginosa*. Photodiagn. Photodyn. Ther. 31:101810. doi: 10.1016/j.pdpdt.2020.101810, PMID: 32437976

[ref133] Perez-Laguna V. Garcia-Luque I. Ballesta S. Perez-Artiaga L. Lampaya-Perez V. Samper S.. (2018). Antimicrobial photodynamic activity of rose Bengal, alone or in combination with gentamicin, against planktonic and biofilm *Staphylococcus aureus*. Photodiagn. Photodyn. Ther. 21, 211–216. doi: 10.1016/j.pdpdt.2017.11.012 29196246

[ref134] Persson G. R. Renvert S. (2014). Cluster of bacteria associated with peri-implantitis. Clin. Implant. Dent. Relat. Res. 16, 783–793. doi: 10.1111/cid.12052, PMID: 23527870

[ref135] Pinheiro S. L. Schenka A. A. Neto A. A. de Souza C. P. Rodriguez H. M. Ribeiro M. C. (2009). Photodynamic therapy in endodontic treatment of deciduous teeth. Lasers Med. Sci. 24, 521–526. doi: 10.1007/s10103-008-0562-2, PMID: 18427873

[ref136] Polke M. Leonhardt I. Kurzai O. Jacobsen I. D. (2018). Farnesol signalling in *Candida albicans* - more than just communication. Crit. Rev. Microbiol. 44, 230–243. doi: 10.1080/1040841X.2017.1337711, PMID: 28609183

[ref137] Pourabbas R. Khorramdel A. Sadighi M. Kashefimehr A. Mousavi S. A. (2023). Effect of photodynamic therapy as an adjunctive to mechanical debridement on the nonsurgical treatment of peri-implant mucositis: A randomized controlled clinical trial. Dent. Res. J. (Isfahan) 20:1. doi: 10.4103/1735-3327.367900 PMID: 36820137 PMC9937927

[ref138] Pourhajibagher M. Bahador A. (2017). Gene expression profiling of fimA gene encoding fimbriae among clinical isolates of *Porphyromonas gingivalis* in response to photo-activated disinfection therapy. Photodiagn. Photodyn. Ther. 20, 1–5. doi: 10.1016/j.pdpdt.2017.08.001, PMID: 28797828

[ref139] Pourhajibagher M. Beytollahi L. Ghorbanzadeh R. Bahador A. (2018a). Analysis of glucosyltransferase gene expression of clinical isolates of *Streptococcus mutans* obtained from dental plaques in response to sub-lethal doses of photoactivated disinfection. Photodiagn. Photodyn. Ther. 24, 75–81. doi: 10.1016/j.pdpdt.2018.09.005 30223081

[ref140] Pourhajibagher M. Chiniforush N. Shahabi S. Palizvani M. Bahador A. (2018b). Antibacterial and Antibiofilm efficacy of antimicrobial photodynamic therapy against Intracanal *Enterococcus faecalis*: an in vitro comparative study with traditional endodontic irrigation solutions. J. Dent. (Tehran) 15, 197–204. PMID: 30405728 PMC6218464

[ref141] Pourhajibagher M. Kazemian H. Chiniforush N. Hosseini N. Pourakbari B. Azizollahi A.. (2018c). Exploring different photosensitizers to optimize elimination of planktonic and biofilm forms of *Enterococcus faecalis* from infected root canal during antimicrobial photodynamic therapy. Photodiagn. Photodyn. Ther. 24, 206–211. doi: 10.1016/j.pdpdt.2018.09.014 30278277

[ref142] Pourhajibagher M. Keshavarz Valian N. Bahador A. (2022). Theranostic nanoplatforms of emodin-chitosan with blue laser light on enhancing the anti-biofilm activity of photodynamic therapy against *Streptococcus mutans* biofilms on the enamel surface. BMC Microbiol. 22:68. doi: 10.1186/s12866-022-02481-6 35246026 PMC8896274

[ref143] Pourhajibagher M. Mahmoudi H. Rezaei-Soufi L. Alikhani M. Y. Bahador A. (2020a). Potentiation effects of antimicrobial photodynamic therapy on quorum sensing genes expression: A promising treatment for multi-species bacterial biofilms in burn wound infections. Photodiagn. Photodyn. Ther. 30:101717. doi: 10.1016/j.pdpdt.2020.101717 32165339

[ref144] Pourhajibagher M. Rokn A. R. Barikani H. R. Bahador A. (2020b). Photo-sonodynamic antimicrobial chemotherapy via chitosan nanoparticles-indocyanine green against polymicrobial periopathogenic biofilms: ex vivo study on dental implants. Photodiagn. Photodyn. Ther. 31:101834. doi: 10.1016/j.pdpdt.2020.101834 32464265

[ref145] Prada I. Mico-Munoz P. Giner-Lluesma T. Mico-Martinez P. Collado-Castellano N. Manzano-Saiz A. (2019). Influence of microbiology on endodontic failure. Literature review. Med. Oral Patol. Oral Cir. Bucal. 24, e364–e372. doi: 10.4317/medoral.22907, PMID: 31041915 PMC6530959

[ref146] Quishida C. C. Carmello J. C. Mima E. G. Bagnato V. S. Machado A. L. Pavarina A. C. (2015). Susceptibility of multispecies biofilm to photodynamic therapy using Photodithazine(R). Lasers Med. Sci. 30, 685–694. doi: 10.1007/s10103-013-1397-z 23912779

[ref147] Rabello D. G. D. Corazza B. J. M. Ferreira L. L. Santamaria M. P. Gomes A. P. M. Martinho F. C. (2017). Does supplemental photodynamic therapy optimize the disinfection of bacteria and endotoxins in one-visit and two-visit root canal therapy? A randomized clinical trial. Photodiagn. Photodyn. Ther. 19, 205–211. doi: 10.1016/j.pdpdt.2017.06.005, PMID: 28619613

[ref148] Renvert S. Roos-Jansaker A. M. Claffey N. (2008). Non-surgical treatment of peri-implant mucositis and peri-implantitis: a literature review. J. Clin. Periodontol. 35, 305–315. doi: 10.1111/j.1600-051X.2008.01276.x, PMID: 18724858

[ref149] Rismanchian M. Nosouhian S. Shahabouee M. Davoudi A. Nourbakhshian F. (2017). Effect of conventional and contemporary disinfectant techniques on three peri-implantitis associated microbiotas. Am. J. Dent. 30, 23–26. PMID: 29178710

[ref150] Ronqui M. R. de Aguiar Coletti T. M. de Freitas L. M. Miranda E. T. Fontana C. R. (2016). Synergistic antimicrobial effect of photodynamic therapy and ciprofloxacin. J. Photochem. Photobiol. B 158, 122–129. doi: 10.1016/j.jphotobiol.2016.02.036 26971277

[ref151] Rosa R. A. D. Santini M. F. Figueiredo J. A. P. Visioli F. Pereira J. R. Vivan R. R.. (2017). Effectiveness of photodynamic therapy associated with irrigants over two biofilm models. Photodiagn. Photodyn. Ther. 20, 169–174. doi: 10.1016/j.pdpdt.2017.10.003, PMID: 29032227

[ref152] Safioti L. M. Kotsakis G. A. Pozhitkov A. E. Chung W. O. Daubert D. M. (2017). Increased levels of dissolved titanium are associated with Peri-Implantitis - A cross-sectional study. J. Periodontol. 88, 436–442. doi: 10.1902/jop.2016.160524, PMID: 27858551

[ref153] Salvi G. E. Fürst M. M. Lang N. P. Persson G. R. (2008). One-year bacterial colonization patterns of *Staphylococcus aureus* and other bacteria at implants and adjacent teeth. Clin. Oral Implants Res. 19, 242–248. doi: 10.1111/j.1600-0501.2007.01470.x, PMID: 18177429

[ref154] Sanches C. V. G. Sardi J. C. O. Terada R. S. S. Lazarini J. G. Freires I. A. Polaquini C. R.. (2019). Diacetylcurcumin: a new photosensitizer for antimicrobial photodynamic therapy in *Streptococcus mutans* biofilms. Biofouling 35, 340–349. doi: 10.1080/08927014.2019.1606907, PMID: 31066298

[ref155] Satala D. Gonzalez-Gonzalez M. Smolarz M. Surowiec M. Kulig K. Wronowska E.. (2021). The role of *Candida albicans* virulence factors in the formation of multispecies biofilms with bacterial periodontal pathogens. Front. Cell. Infect. Microbiol. 11:765942. doi: 10.3389/fcimb.2021.765942, PMID: 35071033 PMC8766842

[ref156] Sathorn C. Parashos P. Messer H. (2007). Antibacterial efficacy of calcium hydroxide intracanal dressing: a systematic review and meta-analysis. Int. Endod. J. 40, 2–10. doi: 10.1111/j.1365-2591.2006.01197.x, PMID: 17209826

[ref157] Scherer K. M. Bisby R. H. Botchway S. W. Parker A. W. (2017). New approaches to photodynamic therapy from types I, II and III to type IV using one or more photons. Anti Cancer Agents Med. Chem. 17, 171–189. doi: 10.2174/1871520616666160513131723 27173966

[ref158] Schwarz S. R. Hirsch S. Hiergeist A. Kirschneck C. Muehler D. Hiller K. A.. (2021). Limited antimicrobial efficacy of oral care antiseptics in microcosm biofilms and phenotypic adaptation of bacteria upon repeated exposure. Clin. Oral Investig. 25, 2939–2950. doi: 10.1007/s00784-020-03613-w, PMID: 33033920 PMC8060176

[ref159] Sculean A. Deppe H. Miron R. Schwarz F. Romanos G. Cosgarea R. (2021). Effectiveness of photodynamic therapy in the treatment of periodontal and Peri-implant diseases. Monogr. Oral Sci. 29, 133–143. doi: 10.1159/000510189 33427227

[ref160] Sedghi L. DiMassa V. Harrington A. Lynch S. V. Kapila Y. L. (2021). The oral microbiome: role of key organisms and complex networks in oral health and disease. Periodontol. 87, 107–131. doi: 10.1111/prd.12393 PMC8457218 34463991

[ref161] Senadheera D. Cvitkovitch D. G. (2008). Quorum sensing and biofilm formation by *Streptococcus mutans*. Adv. Exp. Med. Biol. 631, 178–188. doi: 10.1007/978-0-387-78885-2_12 18792689

[ref162] Shany-Kdoshim S. Polak D. Houri-Haddad Y. Feuerstein O. (2019). Killing mechanism of bacteria within multi-species biofilm by blue light. J. Oral Microbiol. 11:1628577. doi: 10.1080/20002297.2019.1628577, PMID: 31275529 PMC6598489

[ref163] Shetty B. Ali D. Ahmed S. Ibraheem W. I. Preethanath R. S. Vellappally S.. (2022). Role of antimicrobial photodynamic therapy in reducing subgingival oral yeasts colonization in patients with peri-implant mucositis. Photodiagn. Photodyn. Ther. 38:102803. doi: 10.1016/j.pdpdt.2022.102803, PMID: 35288320

[ref164] Shi H. Li J. Peng C. Xu B. Sun H. (2021). The inhibitory activity of 5-aminolevulinic acid photodynamic therapy (ALA-PDT) on *Candida albicans* biofilms. Photodiagn. Photodyn. Ther. 34:102271. doi: 10.1016/j.pdpdt.2021.102271, PMID: 33785444

[ref165] Shrestha A. Hamblin M. R. Anil K. (2014). Photoactivated rose bengal functionalized chitosan nanoparticles produce antibacterial:biofilm activity and stabilize dentin-collagen. Nanomedicine 10, 491–501. doi: 10.1016/j.nano.2013.10.010, PMID: 24200522 PMC3966929

[ref166] Shuping G. B. Orstavik D. Sigurdsson A. Trope M. (2000). Reduction of intracanal bacteria using nickel-titanium rotary instrumentation and various medications. J. Endod. 26, 751–755. doi: 10.1097/00004770-200012000-00022, PMID: 11471648

[ref167] Solarte D. L. G. Rau S. J. Hellwig E. Vach K. Al-Ahmad A. (2022). Antimicrobial behavior and cytotoxicity of Indocyanine green in combination with visible light and water-filtered infrared A radiation against periodontal Bacteria and subgingival biofilm. Biomedicine 10:956. doi: 10.3390/biomedicines10050956, PMID: 35625693 PMC9138561

[ref168] Soundarajan S. Rajasekar A. (2022). Comparative evaluation of combined efficacy of methylene blue mediated antimicrobial photodynamic therapy (a-PDT) using 660 nm diode laser versus erbium-chromium-yttrium-scandium-gallium-garnet (Er, Cr: YSGG) laser as an adjunct to scaling and root planing on clinical parameters in supportive periodontal therapy: A randomized split-mouth trial. Photodiagn. Photodyn. Ther. 39:102971. doi: 10.1016/j.pdpdt.2022.102971 35738551

[ref169] Souza J. G. S. Costa R. C. Sampaio A. A. Abdo V. L. Nagay B. E. Castro N.. (2022). Cross-kingdom microbial interactions in dental implant-related infections: is *Candida albicans* a new villain? iScience 25:103994. doi: 10.1016/j.isci.2022.103994, PMID: 35313695 PMC8933675

[ref170] Spesia M. B. Caminos D. A. Pons P. Durantini E. N. (2009). Mechanistic insight of the photodynamic inactivation of *Escherichia coli* by a tetracationic zinc(II) phthalocyanine derivative. Photodiagn. Photodyn. Ther. 6, 52–61. doi: 10.1016/j.pdpdt.2009.01.003, PMID: 19447372

[ref171] Strazzi-Sahyon H. B. Cintra L. T. A. Nakao J. M. Takamiya A. S. Queiroz I. O. A. Dos Santos P. H.. (2022). Cytotoxicity of root canal irrigating solutions and photodynamic therapy using curcumin photosensitizer. Photodiagn. Photodyn. Ther. 38:102795. doi: 10.1016/j.pdpdt.2022.102795, PMID: 35263668

[ref172] Stuart C. Schwartz S. Beeson T. Owatz C. (2006). *Enterococcus faecalis*: its role in root canal treatment failure and current concepts in retreatment. J. Endod. 32, 93–98. doi: 10.1016/j.joen.2005.10.049, PMID: 16427453

[ref173] Subhadra B. Surendran S. Lim B. R. Yim J. S. Kim D. H. Woo K.. (2020). Regulation of the AcrAB efflux system by the quorum-sensing regulator AnoR in *Acinetobacter nosocomialis*. J. Microbiol. 58, 507–518. doi: 10.1007/s12275-020-0185-2, PMID: 32462488

[ref174] Sztajer H. Szafranski S. P. Tomasch J. Reck M. Nimtz M. Rohde M.. (2014). Cross-feeding and interkingdom communication in dual-species biofilms of *Streptococcus mutans* and *Candida albicans*. ISME J. 8, 2256–2271. doi: 10.1038/ismej.2014.73, PMID: 24824668 PMC4992082

[ref175] Tan Y. Cheng Q. Yang H. Li H. Gong N. Liu D.. (2018). Effects of ALA-PDT on biofilm structure, virulence factor secretion, and QS in *Pseudomonas aeruginosa*. Photodiagn. Photodyn. Ther. 24, 88–94. doi: 10.1016/j.pdpdt.2018.07.005, PMID: 30006320

[ref176] Tanaka M. Mroz P. Dai T. Huang L. Morimoto Y. Kinoshita M.. (2013). Linezolid and vancomycin decrease the therapeutic effect of methylene blue-photodynamic therapy in a mouse model of MRSA bacterial arthritis. Photochem. Photobiol. 89, 679–682. doi: 10.1111/php.12040, PMID: 23311407 PMC3636181

[ref177] Tavares L. J. de Avila E. D. Klein M. I. Panariello B. H. D. Spolidorio D. M. P. Pavarina A. C. (2018). Antimicrobial photodynamic therapy alone or in combination with antibiotic local administration against biofilms of *Fusobacterium nucleatum* and *Porphyromonas gingivalis*. J. Photochem. Photobiol. B 188, 135–145. doi: 10.1016/j.jphotobiol.2018.09.010 30267963

[ref178] Teles F. R. F. Lynch M. C. Patel M. Torresyap G. Martin L. (2021). Bacterial resistance to minocycline after adjunctive minocycline microspheres during periodontal maintenance: A randomized clinical trial. J. Periodontol. 92, 1222–1231. doi: 10.1002/JPER.17-0565, PMID: 33866555

[ref179] Theodoro L. H. Lopes A. B. Nuernberg M. A. A. Claudio M. M. Miessi D. M. J. Alves M. L. F.. (2017). Comparison of repeated applications of aPDT with amoxicillin and metronidazole in the treatment of chronic periodontitis: A short-term study. J. Photochem. Photobiol. B 174, 364–369. doi: 10.1016/j.jphotobiol.2017.08.012 28863395

[ref180] Theuretzbacher U. Piddock L. J. V. (2019). Non-traditional antibacterial therapeutic options and challenges. Cell Host Microbe 26, 61–72. doi: 10.1016/j.chom.2019.06.004, PMID: 31295426

[ref181] Tokubo L. M. Rosalen P. L. de Cássia Orlandi Sardi J. Freires I. A. Fujimaki M. Umeda J. E.. (2018). Antimicrobial effect of photodynamic therapy using erythrosine/methylene blue combination on *Streptococcus mutans* biofilm. Photodiagn. Photodyn. Ther. 23, 94–98. doi: 10.1016/j.pdpdt.2018.05.004, PMID: 29763739

[ref182] Tzach-Nahman R. Mizraji G. Shapira L. Nussbaum G. Wilensky A. (2017). Oral infection with *P. gingivalis* induces peri-implantitis in a murine model: evaluation of bone loss and the local inflammatory response. J. Clin. Periodontol. 44, 739–748. doi: 10.1111/jcpe.12735, PMID: 28453225

[ref183] Ucuncu M. Mills B. Duncan S. Staderini M. Dhaliwal K. Bradley M. (2020). Polymyxin-based photosensitizer for the potent and selective killing of gram-negative bacteria. Chem. Commun. (Camb.) 56, 3757–3760. doi: 10.1039/D0CC00155D, PMID: 32125330

[ref184] Valm A. M. (2019). The structure of dental plaque microbial communities in the transition from health to dental caries and periodontal disease. J. Mol. Biol. 431, 2957–2969. doi: 10.1016/j.jmb.2019.05.016, PMID: 31103772 PMC6646062

[ref185] Van Acker H. Coenye T. (2017). The role of reactive oxygen species in antibiotic-mediated killing of Bacteria. Trends Microbiol. 25, 456–466. doi: 10.1016/j.tim.2016.12.008, PMID: 28089288

[ref186] van de Lagemaat M. Stockbroekx V. Geertsema-Doornbusch G. I. Dijk M. Carniello V. Woudstra W.. (2022). A comparison of the adaptive response of *Staphylococcus aureus* vs. *Streptococcus mutans* and the development of Chlorhexidine resistance. Front. Microbiol. 13:861890. doi: 10.3389/fmicb.2022.861890, PMID: 35694293 PMC9186159

[ref187] Vecchio D. Gupta A. Huang L. Landi G. Avci P. Rodas A.. (2015). Bacterial photodynamic inactivation mediated by methylene blue and red light is enhanced by synergistic effect of potassium iodide. Antimicrob. Agents Chemother. 59, 5203–5212. doi: 10.1128/AAC.00019-15, PMID: 26077247 PMC4538466

[ref188] Vieira C. Gomes A. T. P. C. Mesquita M. Q. Moura N. M. M. Neves M. G. P. M. S. Faustino M. A. F.. (2018). An insight into the potentiation effect of potassium iodide on aPDT efficacy. Front. Microbiol. 9:2665. doi: 10.3389/fmicb.2018.02665, PMID: 30510542 PMC6252324

[ref189] von Ranke N. L. Bello M. L. Cabral L. M. Castro H. C. Rodrigues C. R. (2018). Molecular modeling and dynamic simulations of agglutinin-like family members from *Candida albicans*: new insights into potential targets for the treatment of candidiasis. J. Biomol. Struct. Dyn. 36, 4352–4365. doi: 10.1080/07391102.2017.1417159, PMID: 29241420

[ref190] Wade W. G. (2021). Resilience of the oral microbiome. Periodontol. 86, 113–122. doi: 10.1111/prd.12365, PMID: 33690989

[ref191] Wainwright M. Maisch T. Nonell S. Plaetzer K. Almeida A. Tegos G. P.. (2017). Photoantimicrobials-are we afraid of the light? Lancet Infect. Dis. 17, e49–e55. doi: 10.1016/S1473-3099(16)30268-7, PMID: 27884621 PMC5280084

[ref192] Wall S. (2019). Prevention of antibiotic resistance - an epidemiological scoping review to identify research categories and knowledge gaps. Glob. Health Action 12:1756191. doi: 10.1080/16549716.2020.1756191, PMID: 32475304 PMC7782542

[ref193] Wang Y. Branicky R. Noe A. Hekimi S. (2018). Superoxide dismutases: dual roles in controlling ROS damage and regulating ROS signaling. J. Cell Biol. 217, 1915–1928. doi: 10.1083/jcb.201708007, PMID: 29669742 PMC5987716

[ref194] Wang Y. Li Y. Huang S. Huang J. Huang X. (2023). An easily achievable strategy to promote the penetration of methylene blue into dentinal tubules. Photodiagn. Photodyn. Ther. 41:103237. doi: 10.1016/j.pdpdt.2022.103237, PMID: 36496126

[ref195] Wang H. Li W. Zhang D. Li W. Wang Z. (2019). Adjunctive photodynamic therapy improves the outcomes of peri-implantitis: a randomized controlled trial. Aust. Dent. J. 64, 256–262. doi: 10.1111/adj.12705 31152567

[ref196] Wang Y. Liu B. Grenier D. Yi L. (2019). Regulatory mechanisms of the LuxS/AI-2 system and bacterial resistance. Antimicrob. Agents Chemother. 63:e01186-19. doi: 10.1128/AAC.01186-19 31383657 PMC6761564

[ref197] Wang Y. Xiao S. Ma D. Huang X. Cai Z. (2015). Minimizing concentration of sodium hypochlorite in root canal irrigation by combination of ultrasonic irrigation with photodynamic treatment. Photochem. Photobiol. 91, 937–941. doi: 10.1111/php.12459, PMID: 25892274

[ref198] Warrier A. Mazumder N. Prabhu S. Satyamoorthy K. Murali T. S. (2021). Photodynamic therapy to control microbial biofilms. Photodiagn. Photodyn. Ther. 33:102090. doi: 10.1016/j.pdpdt.2020.102090, PMID: 33157331

[ref199] Wu M. K. van der Sluis L. W. Wesselink P. R. (2003). The capability of two hand instrumentation techniques to remove the inner layer of dentine in oval canals. Int. Endod. J. 36, 218–224. doi: 10.1046/j.1365-2591.2003.00646.x, PMID: 12657148

[ref200] Wu M. Xu L. Cai Z. Huang S. Li Y. Lei L.. (2019). Disinfection of cariogenic pathogens in planktonic lifestyle, biofilm and carious dentine with antimicrobial photodynamic therapy. Photochem. Photobiol. 96, 170–177. doi: 10.1111/php.13161 31483869

[ref201] Wulf H. C. Philipsen P. (2004). Allergic contact dermatitis to 5-aminolaevulinic acid methylester but not to 5-aminolaevulinic acid after photodynamic therapy. Br. J. Dermatol. 150, 143–145. doi: 10.1111/j.1365-2133.2004.05723.x, PMID: 14746630

[ref202] Xu H. Sobue T. Bertolini M. Thompson A. Vickerman M. Nobile C. J.. (2017). *S. oralis* activates the Efg1 filamentation pathway in *C. albicans* to promote cross-kingdom interactions and mucosal biofilms. Virulence 8, 1602–1617. doi: 10.1080/21505594.2017.1326438, PMID: 28481721 PMC5810487

[ref203] Xue D. Zhao Y. (2017). Clinical effectiveness of adjunctive antimicrobial photodynamic therapy for residual pockets during supportive periodontal therapy: A systematic review and meta-analysis. Photodiagn. Photodyn. Ther. 17, 127–133. doi: 10.1016/j.pdpdt.2016.11.011, PMID: 27888165

[ref204] Yamanaka T. Yamane K. Furukawa T. Matsumoto-Mashimo C. Sugimori C. Nambu T.. (2011). Comparison of the virulence of exopolysaccharide-producing *Prevotella intermedia* to exopolysaccharide non-producing periodontopathic organisms. BMC Infect. Dis. 11:228. doi: 10.1186/1471-2334-11-228 21864411 PMC3182146

[ref205] Yang R. Guo S. Xiao S. Ding Y. (2020). Enhanced wound healing and osteogenic potential of photodynamic therapy on human gingival fibroblasts. Photodiagn. Photodyn. Ther. 32:101967. doi: 10.1016/j.pdpdt.2020.101967, PMID: 32835879

[ref206] Yin R. Wang M. Huang Y. Y. Landi G. Vecchio D. Chiang L. Y.. (2015). Antimicrobial photodynamic inactivation with decacationic functionalized fullerenes: oxygen-independent photokilling in presence of azide and new mechanistic insights. Free Radic. Biol. Med. 79, 14–27. doi: 10.1016/j.freeradbiomed.2014.10.514, PMID: 25451642 PMC4721583

[ref207] Yoshida A. Sasaki H. Toyama T. Araki M. Fujioka J. Tsukiyama K.. (2017). Antimicrobial effect of blue light using *Porphyromonas gingivalis* pigment. Sci. Rep. 7:5225. doi: 10.1038/s41598-017-05706-1 28701797 PMC5507902

[ref208] Yuan L. Lyu P. Huang Y. Y. Du N. Qi W. Hamblin M. R.. (2019). Potassium iodide enhances the photobactericidal effect of methylene blue on *Enterococcus faecalis* as planktonic cells and as biofilm infection in teeth. J. Photochem. Photobiol. B 203:111730. doi: 10.1016/j.jphotobiol.2019.111730 31855718 PMC6947667

[ref209] Zhang C. Du J. Peng Z. (2015). Correlation between *enterococcus faecalis* and persistent Intraradicular infection compared with primary Intraradicular infection: A systematic review. J. Endod. 41, 1207–1213. doi: 10.1016/j.joen.2015.04.008, PMID: 26015157

[ref210] Zhang Q. Ma Q. Wang Y. Wu H. Zou J. (2021). Molecular mechanisms of inhibiting glucosyltransferases for biofilm formation in *Streptococcus mutans*. Int. J. Oral Sci. 13:30. doi: 10.1038/s41368-021-00137-1 34588414 PMC8481554

[ref211] Zhao Z. Ma J. Wang Y. Xu Z. Zhao L. Zhao J.. (2021). Antimicrobial photodynamic therapy combined with antibiotic in the treatment of rats with third-degree burns. Front. Microbiol. 12:622410. doi: 10.3389/fmicb.2021.622410, PMID: 33717010 PMC7943878

[ref212] Zhao S. Sun Z. Ye Z. Wang Y. Wang L. Xing L.. (2018). In vitro photodynamic inactivation effects of benzylidene cyclopentanone photosensitizers on clinical fluconazole-resistant *Candida albicans*. Photodiagn. Photodyn. Ther. 22, 178–186. doi: 10.1016/j.pdpdt.2018.04.001, PMID: 29626527

